# Climate change reshapes the potential transmission risk of human alveolar and cystic echinococcosis in western China: a projection based on MaxEnt models

**DOI:** 10.1186/s40249-026-01482-1

**Published:** 2026-09-04

**Authors:** Xu Wang, Quzhen Gongsang, Shijie Yang, Ying Wang, Sha Liao, Guangzhong Shi, Jia Liu, Fang Yan, Xiaojuan Zhang, Baixue Liu, Yan Kui, Yixi Zhang, Chuizhao Xue, Liying Wang, Shuai Han

**Affiliations:** 1 National Institute of Parasitic Diseases, Chinese Center for Disease Control and Prevention; Chinese Center for Tropical Diseases Research; National Key Laboratory of Intelligent Tracking and Forecasting for Infectious Diseases; NHC Key Laboratory on Parasite and Vector Biology; World Health Organization Collaborating Centre for Tropical Diseases, Shanghai, 200025 China; 2 Xizang Autonomous Regional Center for Disease Control and Prevention, National Health Commission (NHC) Key Laboratory of Echinococcosis Prevention and Control, Lhasa, 850000 China; 3 Sichuan Provincial Center for Disease Control and Prevention, Chengdu, 610041 China; 4 Xinjiang Uygur Autonomous Regional Center for Disease Control and Prevention, Urumqi, 830002 China; 5 Qinghai Provincial Institute for Endemic Diseases Prevention and Control, Xining, 811602 China; 6 Ningxia Hui Autonomous Regional Center for Disease Control and Prevention, Yinchuan, 750011 China; 7 Gansu Provincial Center for Disease Control and Prevention, Lanzhou, 730020 China

**Keywords:** Echinococcosis, *Echinococcus*, Climate change, MaxEnt, One Health, Western China

## Abstract

**Background:**

Echinococcosis, a severe zoonotic parasitic disease, imposes a significant health burden in western China, primarily as alveolar (AE) and cystic (CE) forms caused by *Echinococcus multilocularis* (*Em*) and *E. granulosus *sensu lato (*Egsl*), respectively. Its transmission, tied to a complex host cycle, is strongly influenced by climatic factors. This study aimed to model the ecological niches of these parasites, validate predictions with epidemiological data, and project the impact of climate change on future transmission risk geography.

**Methods:**

Utilizing location data of 1247 human cases diagnosed in western China from 1981 to 2016, obtained from the National Echinococcosis Control Program, and contemporary climate data, we developed optimized Maximum Entropy (MaxEnt) models. This involved defining an appropriate spatial resolution, screening 19 bioclimatic variables to select key predictors (7 for *Em*, 6 for *Egsl*), and identifying optimal model parameters. Model performance was evaluated using the Area Under the Curve (AUC) and AUC of 10% Receiver Operating Characteristic (AUCpROC) curve, and validity was assessed via correlation with county-level incidence. Future habitat suitability was projected for multiple periods (2011–2100) under three climate scenarios (SSP-126, SSP-370, SSP-585) generated by 5 global climate models.

**Results:**

The models demonstrated high predictive accuracy (AUC > 0.92; AUCpROC: 0.084 for *Em*, 0.070 for *Egsl*) and a significant positive correlation with incidence (*ρ* > 0.6, *P* < 0.0001). *Em* and *Egsl* exhibited distinct climatic niches: *Em* prefers cool, stable climates with moderate summer moisture, while *Egsl* spreads in areas with dry-cold winters, warm-humid summers, and moderate seasonality. Future projections reveal a spatially heterogeneous reshaping of risk. While overall suitability increases, *Em*'s suitable area is projected to shift toward higher altitudes (e.g., expanding in Qinghai and Xizang but contracting in Ningxia and Gansu). In contrast, *Egsl* shows widespread outward expansion from current endemic margins, particularly across the Tibetan Plateau. Qinghai and Xizang are identified as key expansion hotspots for both *Em* and *Egsl*.

**Conclusion:**

The findings confirm distinct ecological drivers for the suitability of *Em* and *Egsl*, and indicate that climate change will potentially shift the endemic areas of AE and expand those of CE in western China. This underscores the necessity for forward-looking, spatially targeted surveillance and control strategies, informed by a One Health approach, to mitigate the evolving burden of echinococcosis.

**Supplementary Information:**

The online version contains supplementary material available at https://doi.org/10.1186/s40249-026-01482-1.

## Background

Echinococcosis is a zoonotic parasitic disease caused by the larval stages of tapeworms belonging to the genus *Echinococcus* [1]. The two primary forms are alveolar echinococcosis (AE) and cystic echinococcosis (CE), caused by *Echinococcus multilocularis* (*Em*) and *Echinococcus granulosus *sensu lato (*Egsl*), respectively [2]. The life cycle of *Echinococcus* involves definitive hosts (such as dogs, foxes, and other carnivores) and intermediate hosts (such as sheep, cattle, and rodents) [3–5]. Humans, act as accidental hosts, becoming infected through the ingestion of eggs, which leads to the development of chronic lesions primarily in the liver or lungs [6]. The complexity of this transmission cycle makes echinococcosis a significant public health challenge, particularly in economically disadvantaged regions where inadequate healthcare resources and insufficient control measures exacerbate the substantial disease burden [7].

It is estimated that CE accounts for the majority of global echinococcosis cases, with a mortality rate of 2–4% [8]. Although AE is less common, it exhibits high lethality, with a 10-year mortality rate of up to 90% in untreated patients [9]. It is estimated that 2–3 million people are infected with echinococcosis globally, with approximately 200,000 new cases reported annually [10]. According to World Health Organization (WHO) data, the disease causes about 19,300 deaths and 871,000 disability-adjusted life years each year, resulting in annual economic losses exceeding USD 3 billion [11].

China is one of the countries most severely affected by echinococcosis globally [12]. A national epidemiological survey conducted from 2012 to 2016 identified 368 (now corrected to 370) endemic counties in China with confirmed evidence of ongoing *Echinococcus* transmission cycles [13]. These counties are distributed across provincial-level administrative regions (PLARs) in western, northwestern, and northern China, encompassing approximately 166,000 patients and a population at risk exceeding 50 million [14]. PLARs such as Qinghai, Sichuan, Gansu, Ningxia, Xinjiang, and Xizang are co-endemic for both CE and AE [15]. These regions feature diverse geographical environments including the high-altitude meadows of the Tibetan Plateau (Qinghai–Xizang Plateau, encompassing Xizang, Qinghai, and western Sichuan), the agricultural areas of the Loess Plateau (Ningxia), and desert fringes (Xinjiang and Gansu) [16]. These ecosystems support rich populations of host animals (e.g., foxes, dogs, rodents, and livestock), thereby sustaining complex transmission networks of *Echinococcus* and leading to the persistent prevalence of echinococcosis [17].

Since the launch of the National Echinococcosis Control Program (NECP) in 2005, measures such as deworming dogs, managing livestock slaughter, and screening human populations have led to a decline in the prevalence of echinococcosis in most areas. However, localized infections persist, particularly in remote pastoral regions [15].

The prevalence of echinococcosis is significantly influenced by ecological factors, with climate playing a pivotal role [18]. Climatic variables such as temperature and precipitation directly affect the survival and infectivity of *Echinococcus* eggs in the environment and indirectly influence the transmission chain by altering vegetation cover and population dynamics of host species [19]. Studies indicate that *Em* eggs survive longest under low temperatures (−18 °C to 4 °C) and moderate humidity, whereas the optimal survival temperature for *Egsl* eggs ranges from 0 °C to 10 °C [20, 21]. Notably, *Egsl* eggs can remain viable and infectious for up to 41 months in environments characterized by wide temperature fluctuations (−3 °C to 37 ℃) and arid conditions [21]. Climate warming may reduce egg survival rates but could also expand the habitat range of intermediate hosts, such as rodents and ungulates, thereby potentially increasing transmission risks. For example, increased precipitation can improve the quality and abundance of vegetation, which in turn may raise the population density of herbivorous intermediate hosts and indirectly affect the activity patterns of the definitive hosts that prey on them [22, 23]. Additionally, increased rainfall or glacier meltwater runoff due to rising temperatures may wash *Echinococcus* eggs into drinking water reservoirs, elevating the risk of waterborne transmission [24, 25]. These intricate interactions establish climate change as a pivotal driver in the transmission dynamics of *Echinococcus*, potentially elevating the risk of human infection. In particular, global warming may alter the geographical distribution of these parasites, which could lead to new and unforeseen patterns of the diseases they cause [19].

This study employs the Maximum Entropy (MaxEnt) model, integrating historical climate data and future climate scenarios, to systematically analyze the climatic suitability of *Em* and *Egsl* [26]. This well-established species distribution modeling tool, has been widely applied in the assessment and prediction of parasite spatial distribution and the transmission risks of parasitic diseases [27]. For instance, in North America, it has been successfully used to characterize the ecological niche of *Em* and predict a potential significant increase in its future suitable habitat [28]. In Europe, the model has also been utilized to simulate the current and future distribution of the same parasite, identifying a trend of suitable areas shifting towards higher latitudes/altitudes driven by climate warming [29]. These studies validate the effectiveness and reliability of this model in researching parasites with complex life cycles. However, there remains a notable gap in research that comprehensively assesses the niche differentiation between the two primary pathogenic *Echinococcus* species in the highly endemic regions of western China and systematically projects the future evolution of their transmission risk geography. Therefore, building upon an optimized model, this study further explores the association between the ecological niche characteristics of these two parasites and human echinococcosis epidemiological data. It aims to evaluate the differential impact of climate change on the transmission risk and potential geographical distribution of echinococcosis in China and to identify spatial trends in future epidemic risk intensity. The findings are expected to provide a scientific basis for formulating precise, forward-looking, and regionally differentiated prevention and control strategies.

## Methods

### Study area

The core study area was defined as the region in China co-endemic for both AE and CE, based on the NECP database. It encompasses the entire PLARs of Gansu, Ningxia, Xizang, Qinghai, and Xinjiang, as well as the Ganzi, Aba, and Liangshan Autonomous Prefectures and Ya’an City in Sichuan Province [15]. To accurately delineate the species' ecological niches and avoid the spatial niche truncation problem, it is essential to correctly define the modeling background region [30]. Referring to aggregated data from Wang et al. [5], we defined the species' "movement" (M) region (accessible area) as all provincial-level administrative regions on the Asian mainland (excluding islands) where the species has been reported in the literature. This M region was designed to encompass a broader spectrum of environmental conditions historically encountered by the species, thereby enhancing the completeness of the niche estimation [31].

### Case location data collection

We extracted clinical data for echinococcosis patients with initial diagnoses between 1981 and 2016 from the NECP database. All cases were diagnosed and confirmed strictly according to the *Diagnostic Criteria for Echinococcosis (WS 257–2006)* (Supplementary Document 1, https://cspd.ipd.org.cn/standardv81.html). The database records detailed information for each case, including settlement location (at the administrative village level), identifier, occupation, date of first diagnosis, and follow-up records. Each case underwent a four-tier review process by county, city, provincial, and national Centers for Disease Control and Prevention; only reviewed cases were included [15]. Geographic coordinates (longitude and latitude) for the administrative center of the village/community of residence for each case were manually obtained using the Baidu Coordinate Pickup System (https://lbs.baidu.com/maptool/getpoint). The administrative center typically represents the most densely populated area and the location with the highest case reporting density. These coordinate points were used as species presence data for model input. Selecting an appropriate spatial resolution for environmental variable rasters that matches the precision of the presence point data can effectively reduce model prediction bias [31]. Therefore, we downloaded the initial administrative village boundary data for each PLAR within the study area from Lifang Data (https://wxredian.com/art?id=34e311a2e35033b723db183c4febaba4). These data were then further checked and corrected against the 2023 administrative division data from Ruiduobao Map (https://map.ruiduobao.com/). Subsequently, the area of each administrative village was calculated. The normality of the village area dataset was tested using the Kolmogorov-Smirnov test (*α* = 0.05) to determine whether to use the mean (if normal) or the median (if non-normal) as the representative area for guiding the selection of environmental raster resolution (pixel size).

### Acquisition and processing of climate variables​

Climate variables were acquired from the CHELSA (Climatologies at High Resolution for the Earth’s Land Surface Areas) 2.1 database (https://chelsa-climate.org/) [32]. We obtained the original raster data for 19 bioclimatic variables (Bio01–Bio19) with a spatial resolution of approximately 30 arc-seconds (~ 1 km) [33]. Given the potential incubation period of echinococcosis can exceed five years, climate data from 1981 to 2010 were used to represent the "current" baseline period, aiming to more accurately capture the long-term environmental drivers of disease transmission [34]. To predict the impact of future climate change, we acquired data for three future periods (2011–2040, 2041–2070, 2071–2100) under three Shared Socio-economic Pathways (SSPs: SSP-126, SSP-370, SSP-585) simulated by five Global Climate Models (GCMs: GFDL-ESM4, IPSL-CM6A-LR, MPI-ESM1-2-HR, MRI-ESM2-0, UKESM1-0-LL) to account for uncertainty in future climate projections. To improve computational efficiency and match the spatial precision of the case data, original bioclimatic variable rasters were aggregated using an averaging method to an optimal resolution. This resolution was determined to be greater than the average/median village area and to encompass the scale of herders' grazing movements. Subsequently, all aggregated rasters were clipped to the extent of the defined M region using its vector boundary. Then, all coordinate points and subsequent environmental variables were projected to the Krasovsky_1940_Albers coordinate system for consistent spatial analysis.

### Data cleaning

Species presence point coordinates (case locations) were overlaid with the processed environmental variable layers to extract the corresponding values of the 19 variables for each point. To eliminate environmentally redundant points caused by limited raster resolution, the combinations of environmental variable value for all presence points were compared. Points sharing identical environmental variable value combinations but having different spatial coordinates (i.e., located within the same environmental raster cell) were considered "environmental duplicates". To prevent model training bias, only the first point from each unique environmental combination was retained, generating a cleaned presence point dataset using the dplyr package in R [35]. This step ensured that each input point represented a distinct environmental context, enhancing model training efficiency and stability.

### Environmental variable screening

MaxEnt is a widely-used species distribution modeling method that predicts or infers distributions based on incomplete information by determining the probability distribution of maximum entropy [26]. Two parameters that significantly influence the MaxEnt model's predictive outcome are the Feature Combination [FC, including Linear (L), Quadratic (Q), Hinge (H), Product (P), and Threshold (T)] and the Regularization Multiplier (RM) [36]. Therefore, we constructed a 48-parameter grid comprising 6 common FCs (L, LQ, H, LQH, LQHP, LQHPT) and 8 RMs (0.5 to 4, in steps of 0.5) for exploration. First, a Pearson correlation matrix for all 19 bioclimatic variables was calculated based on all presence points. Then, a preliminary MaxEnt model was run once for each parameter combination using the dismo package with all variables. For each run, 10,000 background points were randomly sampled from the M region, with 75% of data for training, 25% for testing, and other MaxEnt parameters kept at default settings. Variable contribution results were extracted via the Jackknife test [37]. Iterative screening followed these rules: (a) retain the variable with the highest contribution; (b) sequentially evaluate subsequent variables in descending order of contribution; if the absolute correlation coefficient with any variable in the selected set exceeded the threshold (|*r*|= 0.7), it was removed to mitigate multicollinearity; otherwise, it was retained [35]. This process generated an optimized variable subset for each parameter combination.

### Model parameter optimization​

Complete parameter optimization was performed for all candidate variable subsets. A spatial block cross-validation (4 folds based on longitude/latitude grids) was employed to test all 48 parameter combinations, calculating the corrected Akaike Information Criterion (AICc) and the test Area Under the Curve (AUCtest) of the Receiver Operating Characteristic (ROC) for each using the ENMeval package. Following Warren et al. [38], the combination with the smallest AICc was selected; if AICc values were identical, the combination with the higher AUCtest was chosen. This criterion aimed to balance prediction accuracy with model parsimony, avoiding overfitting.

### Model construction​ and validation​

Using the selected optimal variable combination and parameters, 100 independent model replicates were run to assess model stability. For each replicate, 10,000 background points were randomly generated, and all point data were randomly partitioned into 75% for training and 25% for testing. This yielded 100 model files and corresponding current climate suitability prediction maps. For each run, the AUCtraining, AUCtest, AUC of Partial ROC Curve (AUCpROC, 10%), and the Continuous Boyce Index (CBI) were calculated [39, 40]. The replicate with the highest AUCpROC value was selected as the primary indicator for the best model, with AUCtest, AUCtraining, and CBI serving as secondary reference indicators, ultimately determining a single best model. We plotted bar charts depicting the percentage contribution and permutation importance of the variables in the optimal model, along with response heatmaps and curves. The continuous suitability probability output (cloglog format) from the best model was visualized as a distribution map. The 10% of the Training Presence (TP10) logistic output value was used as the threshold to delineate suitable and non-suitable areas [41]. Within suitable areas, probability values were divided into three equal intervals to define low, medium, and high suitability zones. The area and proportion of each suitability level within each PLAR were calculated. To epidemiologically validate the model predictions, we calculated the Spearman's rank correlation coefficient between the predicted *Echinococcus* suitability (cloglog value) (from MaxEnt model) and the echinococcosis incidence rate (based on NECP data) across 390 county-level administrative units within the core study area [15]. The incidence rate was calculated as the average annual number of cases per million people for the period 2004–2022 for each county. The predicted suitability for correlation was obtained by summing the cloglog values of all raster cells within each county-level unit. A significant positive correlation (*P* < 0.05) would indicate that the model predictions effectively reflect the spatial risk pattern of the actual disease.

### Future projection and change analysis​

Based on the validated best model and variable set, the future suitability of the two *Echinococcus* species was projected for 45 future climate scenarios (3 periods × 3 SSPs × 5 GCMs) using the dismo package. For each period-SSP combination, the future predicted value for each pixel and its difference from the current baseline simulation were calculated. The mean and standard deviation (*SD*) of predictions from the 5 GCMs were summarized to illustrate the trend of average suitability probability over time for each sub-region (6 PLARs) and visualized in spatial distribution maps. Concurrently, the percentage area of increased, decreased, and unchanged suitability probability within each PLAR was calculated.

Continuous suitability probability maps for the current period and each future scenario were converted to binary maps (suitable/unsuitable) using the TP10 threshold. Through spatial overlay analysis, each pixel was classified into one of four change types: Stable Suitable (S-S), Suitable Gained (S-G), Suitable Lost (S-L), Stable Unsuitable (S-U). The most frequent change type (mode) among the 5 GCM projections for each pixel was assigned as its final change type. In cases where multiple change types shared the highest frequency, the final type was assigned based on the following priority order: S-S, S-L, S-G, S-U. Subsequently, spatial distribution maps of suitability change type were generated, and the percentage area for each change type per PLAR was calculated.

### Model extrapolation risk assessment

To assess the reliability of future predictions in non-analog environments, an extrapolation risk analysis was performed for all 45 future scenarios [42]. Using the environmental variable values from all available presence points as the reference distribution, the Multivariate Environmental Similarity Surface (MESS) analysis was employed to calculate the similarity between each future environmental pixel and the environmental range of the training data [43]. A negative MESS value indicates that the pixel's environmental conditions fall outside the training data range (extrapolation), where prediction uncertainty is higher. The average percentage area and *SD* of extrapolation regions for each future period and SSP were calculated. For each pixel, we counted​ the number of GCMs (out of 5) for which it was predicted as extrapolation (MESS < 0). Extrapolation risk levels were classified: 0 GCMs (low risk), 1–2 GCMs (medium risk), 3–4 GCMs (high risk), 5 GCMs (very high risk). Finally, extrapolation risk maps were generated to provide an important caveat for interpreting future prediction results.

### Analytical tools​

All spatial data processing, modeling, statistical analyses, and visualization were conducted in R 4.5.1 (R Foundation, Vienna, Austria) within the RStudio environment. Map layouts were prepared using ArcMap 10.8 (ESRI Inc., Redlands, CA, USA).

## Results

### Case data​ in western China from 1981 to 2016

**Fig. 1:**
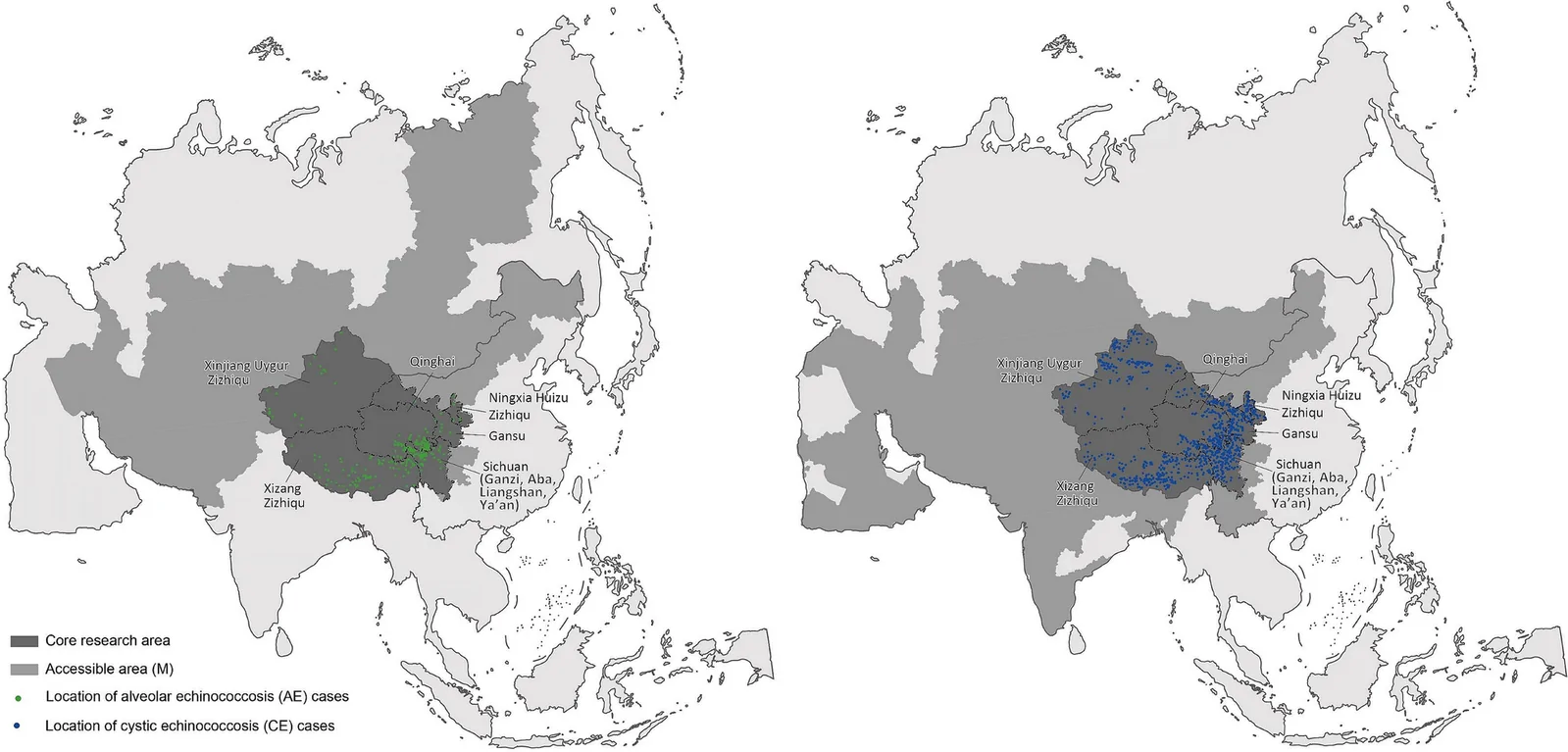
Spatial distribution of 245 alveolar echinococcosis cases (left) and 1002 cystic echinococcosis cases (right) included in this study. Map approval number: GS (2026) 3430

A total of 5363 echinococcosis cases with complete temporal and spatial information were initially identified in western China (Gansu, Ningxia, Xizang, Qinghai, Xinjiang, and partial Sichuan) during the period from 1981 to 2016. After data cleaning, 1247 cases were retained for the MaxEnt model, comprising 245 AE cases and 1002 ce cases (Fig. 1). The study area covered 48,857 administrative villages across six provincial-level regions, with a median village area of 7.10 km^2^ (Q1 = 2.85, Q3 = 19.09). Xizang had the largest median village area at 54.99 km^2^ (Q1 = 22.86, Q3 = 143.49) (Table 1). Considering that the average distance between pastoral settlements and grazing areas on the Tibetan Plateau (in regions with extremely low population density above 4000 m elevation) is 12.34 ± 8.45 km [44], we selected a spatial resolution of approximately 10 arc-minutes (~ 18 km) for the environmental rasters. This coarse resolution ensures that each grid cell (pixel) adequately captures environmental conditions at the village scale, while also helping to absorb the geographic uncertainty introduced by the imperfect match between reported case locations (administrative centers) and potential actual infection sites (grazing areas). Moreover, it is more suitable for continental‑scale spatial modeling and provides more robust spatio‑temporal projections [31].

**Table 1:** Area of administrative villages and number of included cases (1981–2016) in six provincial-level administrative regions (PLARs) within the core study region in western China

Name of PLAR	Area of PLAR (thousand km^2^)	No. of administrative villages/communities	Median area of administrative villages (km^2^)	First quartile (Q1, km^2^)	Third quartile (Q3, km^2^)	No. of included cases (total number of cases)	
						Cystic echinococcosis	Alveolar echinococcosis
Gansu	425.80	16,258	5.94	3.16	11.16	171 (485)	9 (10)
Xizang	1228.40	5265	54.99	22.86	143.49	251 (1078)	81 (136)
Qinghai	722.30	4147	5.58	2.91	17.09	151 (938)	72 (721)
Ningxia	66.40	2180	8.00	3.33	16.67	46 (125)	5 (15)
Xinjiang	1664.90	12,360	1.07	1.07	11.94	197 (484)	20 (32)
Part of Sichuan	309.20	8647	12.60	5.88	35.75	186 (1027)	58 (312)
Total	4417.00	48,857	7.10	2.85	19.09	1002 (4137)	245 (1226)

Part of Sichuan: Ganzi, Aba, and Liangshan Autonomous Prefectures and Ya’an City

### Variable selection and parameter optimization

**Fig. 2:**
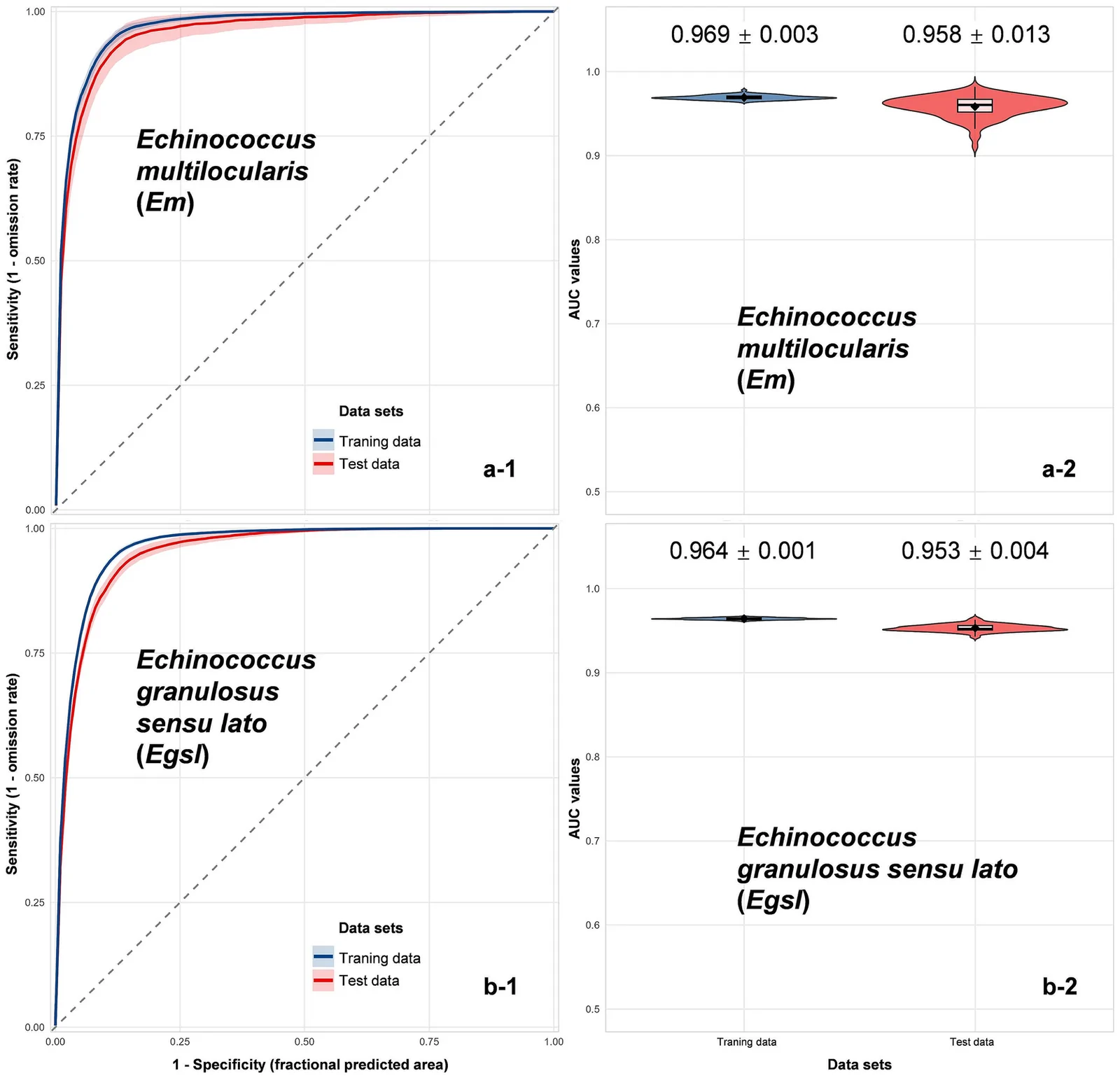
Mean Receiver Operating Characteristic (ROC) curves and Area Under the Curve (AUC) distributions from 100 independent model replicates. **a-1 **and **a-2**: Mean ROC curve and AUC distribution for the *Echinococcus multilocularis* (*Em*) model, respectively. **b-1** and **b-2**: Mean ROC curve and AUC distribution for the *Echinococcus granulosus *sensu lato (*Egsl*) model, respectively

Following preliminary analysis using all 19 bioclimatic variables for each of the 48 parameter combinations (6 FCs × 8 RMs), screening was conducted based on variable correlation (|*r*|≤ 0.7) and contribution assessment (see Supplementary Table S1, S2). For *Em*, ten candidate variable sets meeting the criteria were identified (Supplementary Table S3). After 480 tests (evaluating all 48 parameter combinations for each set), 7 bioclimatic variables were selected for the final model: bio02 (mean diurnal range), bio03 (isothermality), bio07 (temperature annual range), bio10 (mean temperature of warmest quarter), bio15 (precipitation seasonality), bio18 (precipitation of warmest quarter), and bio19 (precipitation of coldest quarter). The optimal parameter combination was LQHPT_1.5 (AICc = 4391.45, AUCtest = 0.97) (Supplementary Table S5). The model for *Em* constructed with this variable and parameter set was run for 100 independent replicate, yielding a mean AUCpROC of 0.072 ± 0.003, AUCtraining of 0.969 ± 0.003, AUCtest of 0.958 ± 0.013 (Fig. 2a), and a CBI of 0.886 ± 0.050. For *Egsl*, two candidate variable sets were identified. After 96 tests, 6 bioclimatic variables were retained: bio02, bio04 (temperature seasonality), bio09 (mean temperature of driest quarter), bio10, bio18, and bio19 (Supplementary Table S4). The optimal parameter combination was LQHPT_0.5 (AICc = 18,964.23, AUCtest = 0.96) (Supplementary Table S6). The corresponding model yielded a mean AUCpROC of 0.066 ± 0.002, AUCtraining of 0.964 ± 0.001, AUCtest of 0.953 ± 0.004 (Fig. 2b), and a CBI of 0.984 ± 0.010. These evaluation metrics indicate that the constructed models possess good stability, discriminatory ability, predictive consistency, and show no signs of severe overfitting.

### Response of environmental variables

**Fig. 3:**
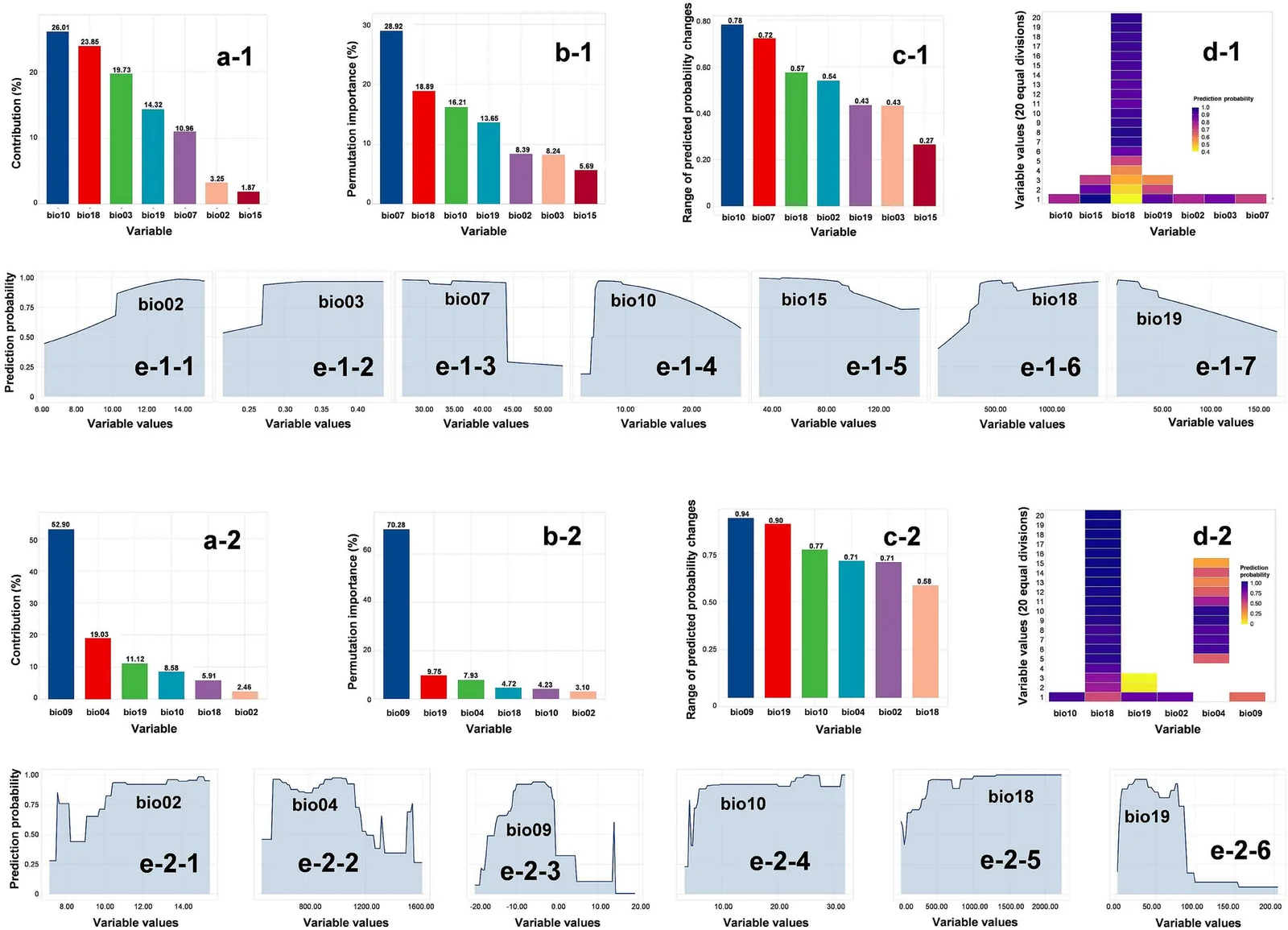
Characteristics of the climatic variables in the optimal models predicting *Echinococcus* suitability. **a-1** and **a-2**: Percent contribution of variables for the *Echinococcus multilocularis* (*Em*) and *Echinococcus granulosus *sensu lato (*Egsl*) models, respectively. **b-1** and **b-2**: Permutation importance of variables for the *Em* and *Egsl* models, respectively. **c-1** and **c-2**: Response range of variables for the *Em* and *Egsl* models, respectively. **d-1** and **d-2**: Heatmaps of variable response magnitude for the *Em* and *Egsl* models, respectively. **e-1-1** to **e-1-7**: Response curves for the seven predictor variables in the *Em* model. **e-2-1** to **e-2-6**: Response curves for the six predictor variables in the *Egsl* model

**Fig. 4:**
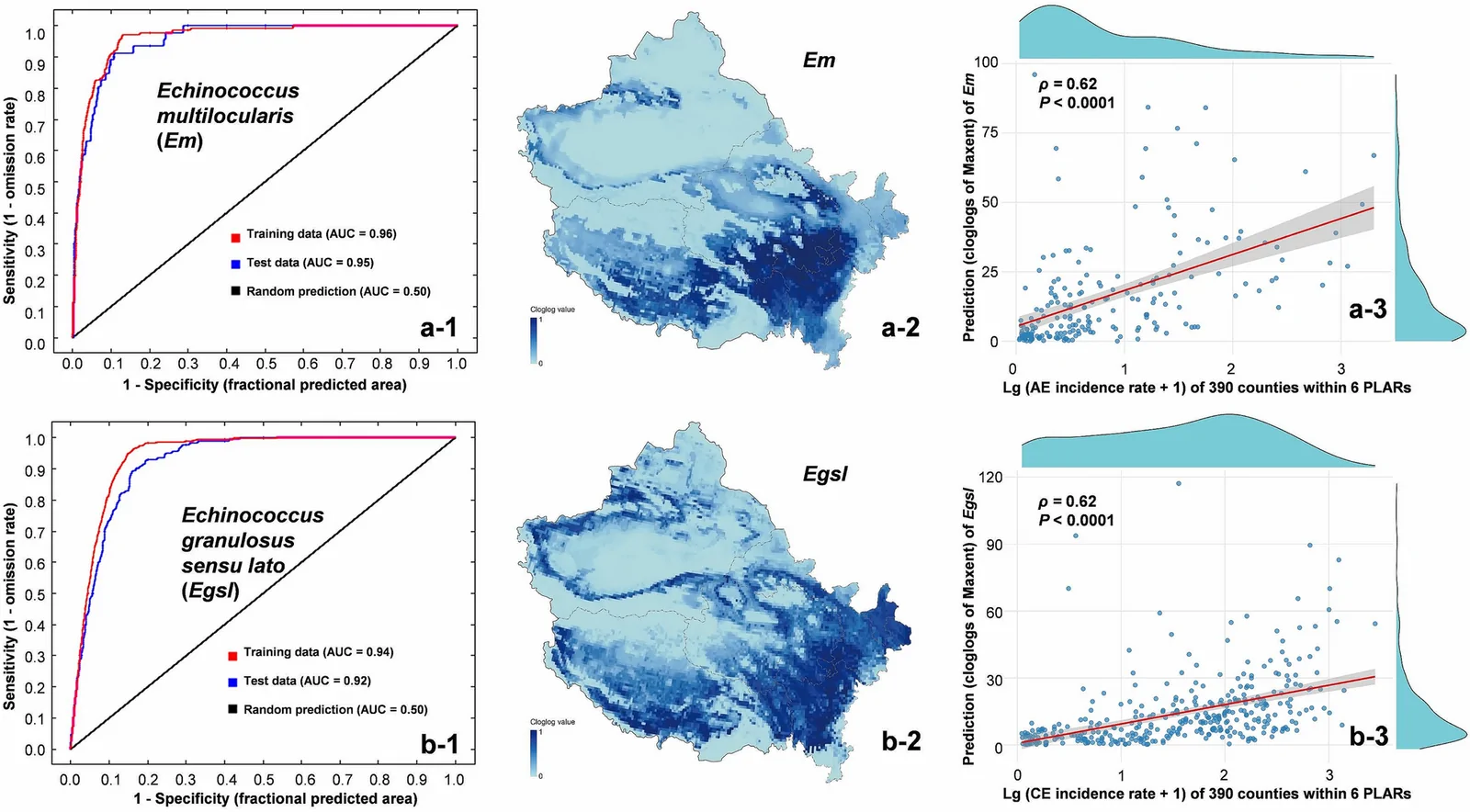
Predictive performance, results, and epidemiological validation of the optimal MaxEnt models for *Echinococcus multilocularis* (*Em*) and *Echinococcus granulosus *sensu lato (*Egsl*), based on the current baseline period (1981–2010) across six provincial-level administrative regions (PLARs). **a-1** and **b-1**: Receiver Operating Characteristic (ROC) curves for the *Echinococcus multilocularis* (*Em*) and *Echinococcus granulosus *sensu lato (*Egsl*) models, respectively; **a-2** and **b-2**: Spatial distribution of predicted suitability for *Em* and *Egsl*, respectively; **a-3** and **b-3**: Correlation between model-predicted suitability (cloglog) for *Em* (top)/*Egsl* (bottom) and the actual average annual incidence of AE (top)/CE (bottom) across 390 county-level units within the study area. Map approval number: GS (2026) 3430

**Fig. 5:**
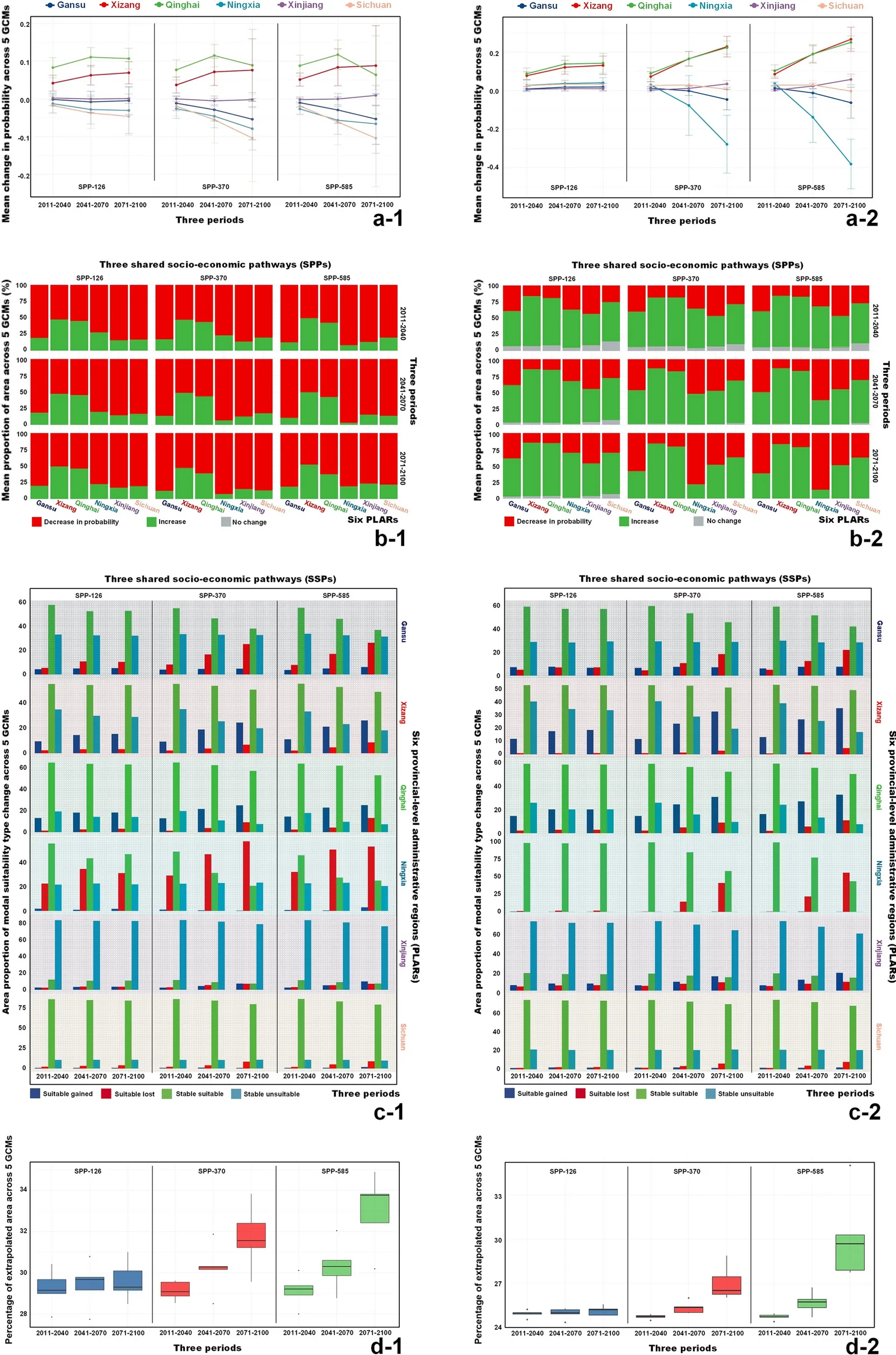
Projected changes in suitability probability, suitability type, and extrapolation for *Echinococcus multilocularis* (*Em*, left) and *Echinococcus granulosus *sensu lato (*Egsl*, right) under three Shared Socio-economic Pathways (SSPs) across future period (2011–2100) based on five Global Climate Models (GCMs). **a-1** and **a-2**:​ Line graphs showing the mean change in habitat suitability probability for *Em* and *Egsl*, respectively; **b-1** and **b-2**:​ Stacked area charts depicting the area percentage of the mean change in habitat suitability probability for *Em* and *Egsl*, respectively; **c-1** and **c-2**:​ Bar charts illustrating the area percentage of projected modal change in habitat suitability type for *Em* and *Egsl*, respectively; **d-1** and **d-2**:​ Distribution charts showing the mean percentage of extrapolated area for *Em* and *Egsl*, respectively

**Fig. 6:**
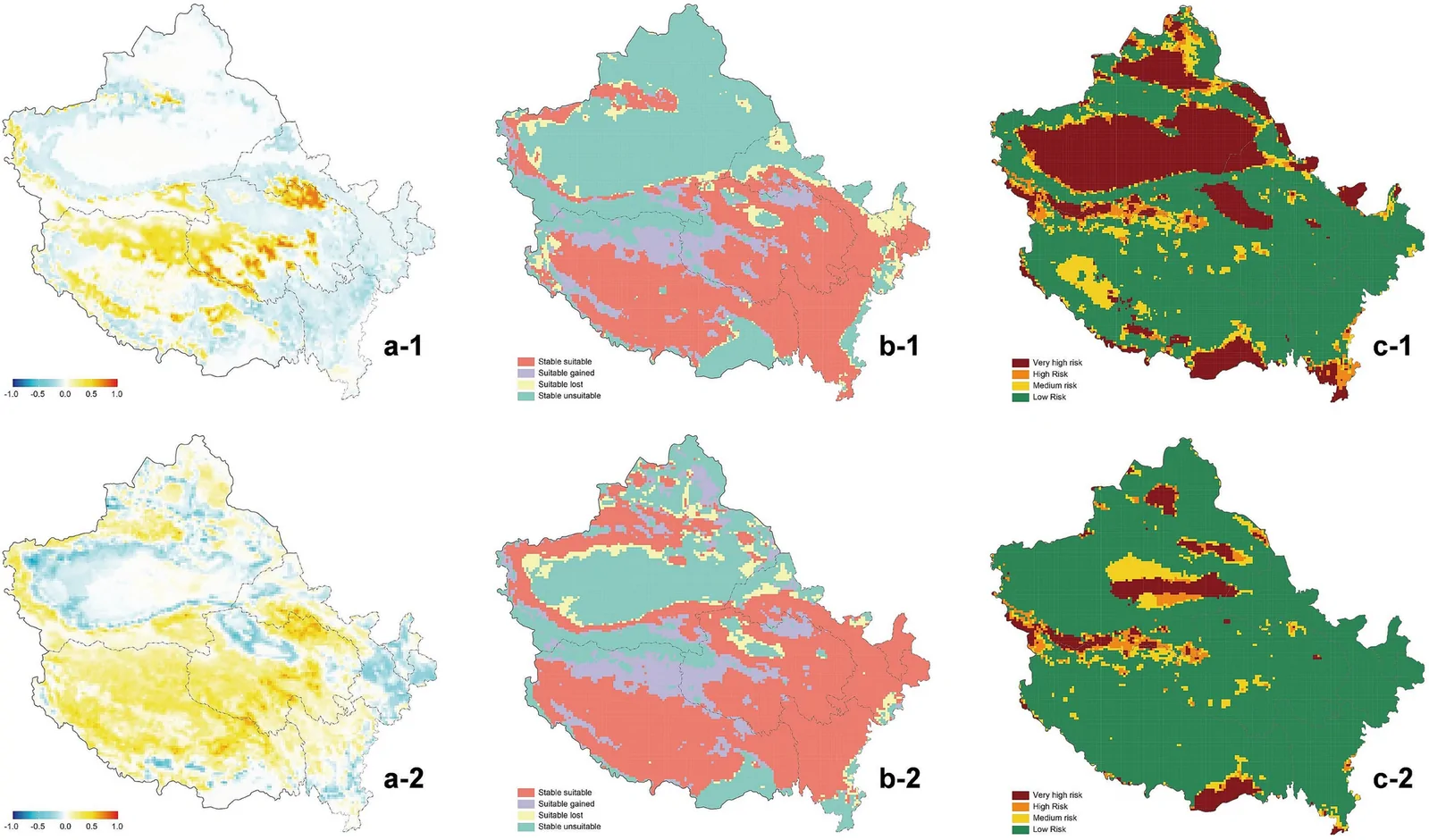
Projected change in suitability probability and suitability type, and predicted extrapolation risk for *Echinococcus multilocularis* (*Em*, top) and *Echinococcus granulosussensu lato* (*Egsl*, bottom) under the Shared Socioe-conomic Pathway (SSP)−370 scenario for future periods (2041–2070), based on five Global Climate Models (GCMs). **a-1** and **a-2**: Maps of mean change in suitability probability for *Em* and *Egsl*, respectively. **b‑1** and **b‑2**:​ Maps of modal change in suitability type for *Em* and *Egsl*, respectively. **c‑1** and **c‑2**:​ Maps of extrapolation risk levels for *Em* and *Egsl*, respectively. Map approval number: GS (2026) 3430

The best *Em* model, selected based on the highest AUCpROC (0.084), showed that bio07 had the highest permutation importance (28.9%) (Fig. 3b-1), indicating that annual temperature fluctuation is the most distinctive limiting factor for *Em* distribution. Suitability probability dropped sharply when bio07 exceeded approximately 43 units (Fig. 3d-1, e-1-3). However, the core driver defining its "thermal boundary" was bio10, which had the highest percent contribution (26.01%) (Fig. 3a-1) and caused the largest change in predicted probability (0.78) (Fig. 3c-1). Probability peaked under cool conditions (approx. 5–10 °C) and then gradually declined with increasing temperature (Fig. 3e-1-4). Furthermore, the key moisture condition supporting its survival within suitable temperature ranges was bio18, which also showed high importance (23.85% contribution, 18.89% permutation importance) (Fig. 3a-1, b-1). Probability showed a positive, saturating correlation with this variable (Fig. 3e-1-6), indicating *Em*'s dependence on summer moisture, with diminishing returns from excessive precipitation. Other variables played auxiliary roles: bio19 showed a weakly negative response (Fig. 3e-1-7); bio02 and bio03 both showed positive responses (Fig. 3e-1-1, e-1-2). Combined with the bio07 result, this suggests a preference for environments with high diurnal temperature variation but stable inter-annual temperatures. Bio15 had the weakest influence. In summary, *Em* is adapted to environments characterized by overall cool conditions, stable inter-annual temperatures, moderate summer precipitation, and tolerance for large diurnal temperature variation, such as continental high-altitude or high-latitude semi-arid climates.

For *Egsl*, the best model (AUCpROC = 0.070) showed that bio09 had an extremely high permutation importance (70.28%) (Fig. 3b-2) and caused the largest probability change (0.94) (Fig. 3c-2). Its response curve was distinctly unimodal (peak around −10 to 0 °C), with probability dropping rapidly above 0 °C, indicating a relatively narrow optimal range for the temperature of the driest (typically winter) quarter (Fig. 3e-2-3). Secondly, bio04 was a significant contributor (19.03%) and an important auxiliary factor (Fig. 3a-2), with a similarly unimodal response curve (Fig. 3e-2-2), suggesting adaptation to moderate temperature seasonality. The response curve for bio10 was monotonically increasing, with probability rising sharply and remaining high above 0 °C, indicating a preference for warm summers (Fig. 3e-2-4). Regarding moisture conditions, bio19 was more critical than bio18 (Fig. 3a-2, b-2): suitability probability was highest at moderate-to-low values of bio19, while the response to bio18 was relatively flat with generally high probability values (Fig. 3d-2, e-2-5, e-2-6). Bio02 had the lowest contribution (2.46%) (Fig. 3a-2). Thus, *Egsl* is adapted to environments with dry-cold winters (but not severely cold) and warm-humid summers, and moderate annual temperature seasonality, typical of continental climates like those found across Eurasia.

### Model construction and epidemiological validation​

The spatial distribution predictions from the best models showed that the AUC (> 0.92) for both species was significantly higher than the random prediction threshold (AUC = 0.5). The CBI (0.81 for *Em*, 0.99 for *Egsl*) was also above the random threshold (CBI = 0), indicating high predictive accuracy and robustness (Fig. 4a-1, b-1). Using the TP10 threshold (0.2 for *Em*, 0.3 for *Egsl*) to delineate suitable areas, the total proportion of suitable habitat was similar for both (47.75% for *Em*, 47.23% for *Egsl*), but their internal composition differed. *Em*'s suitable area was predominantly of low suitability (25.03%), while *Egsl*'s was mainly of medium and low suitability (combined 35.53%). Spatially, western Sichuan was a common highly suitable area for both *Em* and *Egsl*, with high-suitability area proportions reaching 45.10% and 50.40%, respectively. However, Qinghai was another highly suitable area for *Em* (25.94% high suitability), while Ningxia was an extremely suitable area for *Egsl* (combined medium and high suitability > 93%). Gansu showed widespread suitability for both parasites, but with a higher proportion of medium and high suitability for *Egsl*. Xizang had a relatively wide suitable range but a relatively low proportion of high-suitability area; Xinjiang had the lowest suitability for both parasites (Table 2). At the county level, a significant positive Spearman's rank correlation (*ρ* > 0.6, *P* < 0.0001) (Fig. 4a-3, b-3) was found between the model-predicted parasite suitability probability (Fig. 4a-2, b-2) and the corresponding actual county-level echinococcosis incidence [15], providing epidemiological validation for the model predictions.

**Table 2:** Percentage area of suitable habitat for *Echinococcus multilocularis* (*Em*) and *Echinococcus granulosus *sensu lato (*Egsl*) in six provincial-level administrative regions (PLARs) during the current baseline period (1981–2010), based on the optimal model

Species	Names of the PLARs	Percentage of area				
		Unsuitable area	Suitable area			
			low	Medium	High	Sub-total
*Em*	Gansu	35.71	46.75	6.75	10.79	64.29
	Xizang	41.16	29.10	16.78	12.96	58.84
	Qinghai	29.20	29.24	15.61	25.94	70.79
	Ningxia	20.66	78.87	0.47	0	79.34
	Xinjiang	84.90	12.61	2.36	0.12	15.09
	Part of Sichuan	10.33	21.96	22.61	45.10	89.67
	Total	52.25	25.03	10.41	12.31	47.75
*Egsl*	Gansu	33.99	16.36	31.12	18.53	66.01
	Xizang	46.90	21.48	20.64	10.98	53.10
	Qinghai	38.28	28.29	21.96	11.47	61.72
	Ningxia	0	6.29	57.69	36.01	99.99
	Xinjiang	77.42	12.79	8.23	1.56	22.58
	Part of Sichuan	21.01	6.72	21.88	50.40	79.00
	Total	52.77	17.52	18.01	11.70	47.23

### Changes in future suitability probability

In future scenario projections for *Em*, the trend of change in average suitability probability across PLARs was generally consistent across the three future periods under the three SSPs (Fig. 5a-1). The mean suitability probability (cloglog) for the entire core study region was 0.29–0.30, representing an average increase of 0.02–0.03 compared to the current baseline (1981–2010). However, the area with increased probability was generally smaller than the area with decreased probability, approximately half the latter (Table 3). The mean change was positive in Qinghai and Xizang (especially Qinghai), with areas of increase and decrease being roughly balanced. For instance, under the SSP-370 scenario for the period 2041–2070, the proportions of area with increased and decreased suitability in Xizang are 48.41% and 51.59%, respectively, while in Qinghai the corresponding proportions are 43.11% and 56.88% (all specific figures cited below in this subsection refer to this period and scenario). This suggests an overall improvement in environmental suitability for *Em* in these regions. In contrast, the area of probability decrease (87.39%) in Xinjiang was significantly larger than the area of increase (12.61%), although its mean suitability probability fluctuated near the baseline level. Furthermore, the mean change in suitability probability was negative in Gansu, Ningxia, and especially Sichuan, with a more pronounced declining trend over time and areas of decrease significantly outnumbering areas of increase (Gansu: 13.59% increase vs. 86.41% decrease; Ningxia: 6.69% increase vs. 93.31% decrease; Sichuan: 17.53% increase vs. 82.47% decrease), suggesting a weakening of overall environmental suitability for *Em* in these regions (Fig. 5a-1, b-1, Supplementary Table S7). Considering that the global average surface temperature in 2024 has already exceeded the 1.5 °C threshold above pre-industrial levels set by the Paris Agreement, the warming projected under the SSP‑126 scenario may appear somewhat conservative [45]. Moreover, the present already falls within the 2011–2040 period. Meanwhile, results across climate models for the 2071–2100 period and under the SSP‑585 scenario exhibit considerable uncertainty, showing the largest standard deviation in probability changes. Therefore, the study focuses on presenting the predicted suitability maps for the 2041–2070 period under the SSP-370 scenario (Fig. 6). Under this SSP, areas with stable or slightly increased suitability for *Em* were scattered in a mosaic across most of Xizang, northeastern and southwestern Qinghai, and high-altitude areas and the Ili River Valley of Xinjiang.

**Table 3:** Projected habitat suitability of *Echinococcus multilocularis* (*Em*) and *Echinococcus granulosus *sensu lato (*Egsl*) under three Shared Socio-economic Pathways (SSPs) across future period (2011–2100), based on five Global Climate Models (GCMs)

Species	SSPs	Periods	Suitability probability value				Area percentage of suitability probability change (mean)		Area percentage of suitability type change (mode)				Area percentage of extrapolation
			Mean	*SD*	Mean change	*SD*	Increase	Decrease	S-U	S-L	S-G	S-S	Mode
*Em*	SSP-126	**2011–2040**	0.29	0.03	0.02	0.03	35.47	64.53	47.34	2.61	5.34	44.70	31.22
		**2041–2070**	0.30	0.04	0.03	0.04	33.86	66.14	44.71	4.39	7.97	42.92	30.58
		**2071–2100**	0.30	0.05	0.03	0.05	36.96	63.04	44.39	3.59	8.30	43.72	30.87
	SSP-370	**2011–2040**	0.29	0.03	0.02	0.03	32.90	67.10	47.62	2.91	5.06	44.41	31.33
		**2041–2070**	0.30	0.05	0.03	0.05	30.48	69.52	43.06	5.78	9.63	41.53	31.06
		**2071–2100**	0.29	0.08	0.02	0.08	28.52	71.48	39.74	8.19	12.95	39.12	32.71
	SSP-585	**2011–2040**	0.30	0.03	0.02	0.03	31.77	68.23	46.68	3.39	6.01	43.92	31.02
		**2041–2070**	0.30	0.05	0.03	0.05	31.53	68.47	41.59	6.04	11.10	41.27	31.25
		**2071–2100**	0.29	0.10	0.02	0.10	34.87	65.13	38.17	9.28	14.52	38.03	39.34
*Egsl*	SSP-126	**2011–2040**	0.38	0.04	0.04	0.04	70.79	29.18	36.89	3.26	6.76	53.09	11.61
		**2041–2070**	0.40	0.05	0.06	0.05	71.00	28.99	36.68	3.50	6.97	52.85	11.77
		**2071–2100**	0.41	0.06	0.07	0.06	70.92	29.08	36.12	3.53	7.53	52.81	11.37
	SSP-370	**2011–2040**	0.38	0.04	0.04	0.04	70.05	29.89	33.30	4.40	10.36	51.95	10.28
		**2041–2070**	0.41	0.06	0.08	0.06	67.34	32.66	31.09	5.67	12.57	50.68	9.85
		**2071–2100**	0.44	0.08	0.10	0.08	63.42	36.58	29.21	6.30	14.44	50.04	10.24
	SSP-585	**2011–2040**	0.38	0.04	0.04	0.04	70.67	29.30	33.47	4.08	10.18	52.27	10.70
		**2041–2070**	0.43	0.06	0.09	0.06	67.95	32.05	27.09	7.72	16.57	48.63	11.71
		**2071–2100**	0.46	0.09	0.12	0.09	61.56	38.44	24.88	8.34	18.77	48.00	21.79

Five GCMs: GFDL-ESM4, IPSL-CM6A-LR, MPI-ESM1-2-HR, MRI-ESM2-0 and UKESM1-0-LL

*SD* Standard Deviation. S-U Stable Unsuitable; S-L Suitable Lost; S-G Suitable Gained; S-S Stable Suitable

In stark contrast, future projections for *Egsl* showed a mean suitability probability (cloglog) of 0.38–0.46, representing an average increase of 0.04–0.12 from the baseline (Table 3). The area with increased probability was generally larger than the area with decreased probability, approximately double the latter. Consequently, under all three SSPs, suitability probability showed significant positive growth (mean > 0, area of increase > area of decrease) in all PLARs except Ningxia, with the most dramatic increases in Qinghai and Xizang (Xizang: 74.75% increase vs. 18.33% decrease; Qinghai: 74.52% increase vs. 18.37% decrease) (Fig. 5a-2, b-2, Supplementary Table S8). Spatial pattern predictions under SSP-370 indicated that areas of increased probability for *Egsl* formed large, contiguous expanses, covering almost the entire Tibetan Plateau and the agro-pastoral ecotone of Xinjiang. Areas of decreased probability were mainly distributed in the southeastern Tibetan Plateau, southeastern Gansu, most of Ningxia, and the fringes of the Tarim Basin in Xinjiang (Fig. 6).

### Changes in future suitability types​

For *Em*, the areas of S-U and S-S each generally remained at approximately 40% from 2011 to 2100. The area of S-L was consistently smaller than that of S-G, typically about half or slightly more than half (Table 3). For *Egsl*, the areas of S-U and S–S were generally maintained at around 30% and 50%, respectively. Its S-L area was also smaller than the S-G area, usually about half or slightly less than half (Table 3). Specifically, influenced by climate change, the suitable area for both species is projected to expand further in Xizang and Qinghai. The S-G area will be markedly larger than the S-L area, and this trend becomes increasingly pronounced under more extreme SSP scenarios and over time. Conversely, the suitable area for *Em* is projected to contract in Gansu and Ningxia, where the S-G area will be far smaller than the S-L area. For *Egsl*, the change magnitude in Gansu and Ningxia is relatively small under the SSP-126 scenario during 2011–2040. However, during 2041–2100, especially under the SSP-370 scenario, the S-L area will become significantly greater than the S-G area; this trend is more pronounced in Ningxia. In the future, most of Xinjiang will remain S-U for both species, often with the S-G area being roughly equal to or slightly larger than the S-L area. Sichuan will remain S-S, but typically with the S-G area being roughly equal to or slightly smaller than the S-L area. The magnitude of change in suitability types for both species in these two PLARs (Xinjiang and Sichuan) is relatively small (Fig. 5c-1, c-2). Spatial distribution maps further reveal detailed change patterns: the suitable area for *Em* shows a distinct trend of shifting towards higher altitudes and latitudes, whereas *Egsl* exhibits a trend of expansion outward from the margins of current endemic areas (Fig. 6).

### Model extrapolation risk analysis

The extent and intensity of model extrapolation increased and intensified with stronger radiative forcing (SSP-126 < SSP-370 < SSP-585) and over time (2011–2040 < 2041–2070 < 2071–2100). This indicates that under higher emission scenarios and more distant future timeframes, the divergence between future and current climates is greater, and the overall uncertainty of model predictions increases accordingly (Table 3, Fig. 5d-1, d-2). Specifically, the extrapolation area for *Em* predictions was relatively high, exceeding 30% of the entire study region (Table 3), primarily concentrated in extreme climate zones such as deserts and high mountains: the Taklimakan Desert in southern Xinjiang, the Gurbantunggut Desert in northern Xinjiang, the Kunlun Mountains area at the Xinjiang-Xizang border, the Qaidam Basin desert in northwestern Qinghai, and the subtropical and tropical monsoon climate zones of southeastern Xizang and the Hengduan Mountains in southwestern Sichuan (Fig. 6). The extrapolation area for *Egsl* was lower, approximately 10% (Table 3), with locations similar to but more limited in area than those for *Em* (Fig. 6), reflecting *Egsl*'s broader adaptation to environmental variables. Caution is warranted when interpreting future predictions for these high-uncertainty areas. Importantly, most of these high extrapolation-risk areas are not concentrated within current *Echinococcus* suitable habitats or traditional echinococcosis endemic regions (Fig. 4a-2, b-2).

## Discussion

This study integrated nationwide epidemiological data on echinococcosis, environmental variables, and species distribution modeling to systematically assess the potential spatial distribution of *Em* and *Egsl* across six PLARs in western China. It represents the first effort to validate model predictions against county-level incidence data and to quantify the evolving transmission risks of *Echinococcus* under future climate scenarios. The results reveal marked differences in environmental drivers, niche requirements, and future risk trajectories between *Em* and *Egsl*, providing a critical scientific foundation for understanding their transmission dynamics and developing differentiated control strategies.

Although *Em* and *Egsl* share certain environmental predictors, such as bio02, bio10, bio18, and bio19, their response patterns to key climatic variables differ substantially. *Em* exhibits a preference for cool climates with moderate summer moisture. In contrast, *Egsl* spreads under conditions of relatively warm-humid summers, dry-cold winters, and moderate seasonality. These findings are consistent with global epidemiological observations: *Em* (and thus AE) is typically endemic in high-latitude or high-altitude regions of the Northern Hemisphere (e.g., the Tibetan Plateau, Central Asia, and Europe), while the distribution of *Egsl* (and CE) is associated with broader continental climate zones (e.g., across Eurasia and South America) [46]. This niche differentiation closely corresponds to differences in the intermediate hosts involved in their life cycles. The primary intermediate hosts of *Em* are voles and other rodents that inhabit relatively cool plateau or meadow environments [47], whereas *Egsl* commonly infects livestock (such as cattle and sheep), whose distribution is closely tied to agricultural activities in valley and agro-pastoral regions with more favorable thermal and hydrological conditions [48]. Thus, the environmental preferences identified by the models reflect not only the physiological constraints on parasite survival but also the outcomes of complex interactions among parasites, their hosts, and local ecosystems [19]. Furthermore, compared to the small mammal hosts of *Em*, the intermediate hosts of *Egsl* (ruminants) can utilize vegetation across a wider range of soil types, which indirectly confers a broader ecological niche to *Egsl*, consistent with its more generalized variable response curves [49]. Other geographical factors, such as soil water retention capacity and organic matter content, may also indirectly modulate transmission by affecting egg survival and regulating vegetation dynamics in intermediate host habitats [50]. These dimensions warrant more comprehensive mechanistic exploration in future studies.

The high predictive performance of the models (evidenced by high AUCpROC, AUCtraining, AUCtest, CBI values) and the strong positive correlation between predicted parasite suitability and actual echinococcosis incidence empirically validate the applicability and reliability of the MaxEnt model in this context [35]. This supports the feasibility of utilizing long-term climatic and environmental variables within a niche-based framework and the maximum entropy principle to forecast parasite and parasitic disease distributions [27, 31, 51]. Notably, the models not only captured known endemic areas but also identified regions with currently underreported yet high potential risk [52]. Such environmentally based risk inference provides clear geographical targets for proactive surveillance and early warning, enabling a shift from passive response to proactive prevention in public health strategies [53].

Projections under future climate scenarios clearly indicate that climate change will exert profound and complex impacts on the epidemic risks of echinococcosis [54]. Although the overall increase in transmission risk and areal expansion of *Echinococcus* are consistent with global warming trends, the significant spatial heterogeneity merits special attention [52]. The marked shift of *Em* risk toward higher altitudes (e.g., in Qinghai and Xizang) may be attributed to warming conditions that improve habitat suitability for rodent intermediate hosts and enhance egg survival [29]. Conversely, in certain current endemic areas such as Ningxia, future climate conditions could exceed the optimal niche of *Em* (e.g., becoming too warm or arid), resulting in a significant risk reduction [55]. In contrast, *Egsl* risk areas exhibit an outward expansion from existing core zones, particularly in PLARs such as Xizang, Qinghai and Xinjiang, potentially due to climate-induced vegetation shifts that facilitate the extension or relocation of grazing areas [56]. Additionally, the persistently high (> 70%) and stable proportion of continuously suitable area for both *Em* and *Egsl* in western Sichuan indicates that this region's geographic and climatic conditions will continue to provide a relatively stable suitable environment over the coming decades [44, 57]. Therefore, this region should be established as a national core area for long-term control and research. These projections emphasize that climate change will not uniformly intensify transmission risk but will drive a structural reconfiguration of the *Echinococcus* transmission landscape [51]. Accordingly, resource allocation for disease control must be forward-looking and adaptive. Regions identified as high-risk expansion zones (e.g., Qinghai, Xizang, and parts of Xinjiang) require enhanced monitoring and intervention, whereas regions with projected declining risk should remain under surveillance to prevent potential resurgence. In stable core suitable areas like western Sichuan, long-term and stable resource investment is essential, serving as a base for in-depth research on key technologies to block transmission chains, ultimately contributing to the goals outlined in the WHO's road map for neglected tropical diseases [58].

These findings further underscore the urgency and importance of adopting a One Health approach for echinococcosis control [59]. Projections indicate that climate change will alter environmental conditions, dynamically impacting the survival and distribution of pathogens, intermediate hosts, and definitive hosts, thus reshaping the spatial risk landscape [60]. Given this complexity, isolated efforts by any single sector are unlikely to achieve sustained success [61]. Therefore, in high-risk expansion regions, multidimensional interventions should be implemented concurrently, including serological screening and health education in human populations, deworming of domestic dogs, livestock vaccination or quarantine (for CE), and wildlife population and habitat management (for AE) [62]. Establishing an integrated, cross-sectoral platform that links human, animal, and environmental health is therefore essential for effectively addressing the evolving challenge of echinococcosis amid climate change [63].

Furthermore, the extrapolation analysis conducted in this study reveals spatiotemporal patterns in model prediction uncertainty. In the near term (2011–2040), the proportion of area with extrapolation risk is relatively low across all SSPs, indicating that predictions within this timeframe are based on relatively familiar environmental space and are more reliable. However, as the projection period extends to the mid- and long-term, the proportion of extrapolation risk area increases systematically. This is because the cumulative effects of long-term climate change drive key environmental factors increasingly outside the range of historical observations, leading the model to encounter "non-analog" environments over more geographical space, thereby increasing prediction uncertainty [64]. Secondly, extrapolation risk is positively correlated with the severity of the climate scenario. For the same future period, SSP-126 corresponds to the lowest extrapolation risk, while SSP-585 leads to the highest extrapolation risk [65]. This result underscores the fundamental importance of climate change mitigation for maintaining the credibility of ecological forecasts: a more moderate future climate path not only implies milder ecological impacts but also allows for greater confidence in predicting those impacts [66]. The extrapolation risk analysis clearly indicates that long-term distribution predictions for *Echinococcus*, particularly *Em*, should be interpreted primarily as early-warning signals indicating the direction of potential change and areas of concern, rather than precise distribution maps [35]. The inherent "prediction uncertainty" must be incorporated into decision-making when formulating long-term, spatially explicit adaptation strategies [42].

Finally, several limitations of this study warrant acknowledgment. First, constrained by data accessibility, the model did not comprehensively incorporate dynamic covariates such as intermediate host population density, livestock movement, wildlife conservation policies, human behaviors, or spatial variations in the coverage and intensity of control programs. These omissions may introduce uncertainty into long-term projections [48, 54]. Second, species distribution models are inherently correlational and do not directly establish causality. For instance, the ecological pathways underlying the association between climate and parasite suitability or disease risk require further elucidation through field studies and controlled experiments. Furthermore, the models assume niche conservatism, meaning that parasite-environment relationships remain constant over time, a premise that may not hold if the parasites adapt to changing conditions [67, 68]. Future research should seek to integrate multi-source dynamic data (such as remotely sensed vegetation dynamics, host population surveys, drug resistance and genetic variation of parasites) and explore coupling with process-based mechanistic models to improve predictive accuracy and strengthen the scientific basis for targeted One Health ​strategies aimed at interrupting echinococcosis transmission [31, 69].

## Conclusions

This study demonstrates a significant positive correlation between the ecologically predicted distribution of *Echinococcus* spp. and the actual incidence of echinococcosis, validating the effectiveness of environmental niche modeling for the spatial risk assessment of the disease. The findings illustrate significant niche differentiation between *Em* and *Egsl*, which underpins their distinct geographical distributions. This approach offers an effective tool for identifying emerging high-risk regions that may not yet be evident from current surveillance data, thereby enabling proactive public health responses.

Climate change is a key driver reshaping the suitable habitats of *Echinococcus* spp. in China, directly influencing the epidemic risks of echinococcosis. Under future climate scenarios, the potential endemic areas for both *Em* (causing AE) and *Egsl* (causing CE) are projected to undergo geographically reconfigurative expansions; however, this trend displays strong spatial heterogeneity. Effectively addressing this evolving challenge necessitates forward-looking, dynamic, and spatially targeted surveillance and control strategies. The complex interactions among climate change, biological factors, and human activities highlighted in this study strongly confirm the necessity of a One Health approach. Future efforts must integrate human health services, veterinary medicine, and environmental management to alleviate the growing disease burden and achieve sustainable control.

## Supplementary Information

Additional file 1

Additional file 2

Additional file 3

Additional file 4

Additional file 5

Additional file 6

Additional file 7

Additional file 8

Additional file 9

## Data Availability

The data is confidential. To gain access, data requestors will need to sign a data access agreement, upon reasonable request. Proposals should be directed to the corresponding authors.

## Abbreviations

AE: Alveolar echinococcosis
AUC: Area under the curve
CE: Cystic echinococcosis
CHELSA: Climatologies at High Resolution for the Earth’s Land Surface Areas
*Egsl*: *Echinococcus granulosus *sensu lato
*Em*: *Echinococcus multilocularis*
FC: Feature combination
GCM: Global climate model
H: Hinge
L: Linear
MaxEnt: Maximum entropy
MESS: Multivariate environmental similarity surface
NECP: National Echinococcosis Control Program
P: Product
PLAR: Provincial-level administrative region
Q: Quadratic
RM: Regularization multiplier
ROC: Receiver operating characteristic
*SD*: Standard deviation
S-L: Suitable lost
S-G: Suitable gained
S-S: Stable suitable
SSP: Shared socio-economic pathway
S-U: Stable unsuitable
T: Threshold
TP10: 10% of the training presence
WHO: World Health Organization
