# Supplementary material for: Climate change reshapes the potential transmission risk of human alveolar and cystic echinococcosis in western China: a projection based on MaxEnt models
Source: Infect Dis Poverty. 2026 Sep 4;15:97. doi: 10.1186/s40249-026-01482-1 (PMC13543363; doi:10.1186/s40249-026-01482-1)
Supplement: Supplementary file 1 [file 40249_2026_1482_MOESM1_ESM.pdf]

**ICS 11.020**

**C59**

**Record No.: 17595-2006**

# WS

**Health Industry Standard of the People's Republic of China**

**WS 257-2006**

# Diagnostic Criteria for Echinococcosis

**Issued: 2006-04-07**

**Implemented: 2006-12-01**

**Issued by the Ministry of Health of the People's Republic of China**

# Foreword

This standard is formulated on the basis of GB 17013-1997 "Diagnostic Criteria and Treatment Principles for Hydatid Disease", and GB 17013-1997 is hereby abolished.

Appendices B, C, and D of this standard are normative appendices, and Appendix A is an informative appendix.

This standard is proposed by the National Technical Committee for Standardization of Endemic and Parasitic Diseases.

This standard is approved by the Ministry of Health of the People's Republic of China.

Drafting organizations of this standard: National Institute of Parasitic Diseases, Chinese Center for Disease Control and Prevention; Xinjiang Uygur Autonomous Region Clinical Institute of Hydatid Disease; Qinghai Institute for Endemic Disease Prevention and Control; Sichuan Provincial Center for Disease Control and Prevention; Xinjiang Uygur Autonomous Regional Center for Disease Control and Prevention.

Main drafters of this standard: Wu Weiping, Wen Hao, Wang Hu, Yang Wen, Tong Suxiang, Jiang Li.

# Diagnostic Criteria for Echinococcosis

**1 Scope**

1.1 This standard specifies the diagnostic basis, diagnostic principles, diagnostic criteria, and differential diagnosis for cystic echinococcosis and alveolar echinococcosis.

1.2 This standard is applicable to the diagnosis of both types of echinococcosis by disease prevention and control agencies and medical institutions at all levels.

**2 Terms and Definitions**

The following terms and definitions apply to this standard.

2.1 **Hydatidosis / Hydatid Disease**

This is the common name for echinococcosis, a zoonotic parasitic disease caused by the larval stages of tapeworms of the genus *Echinococcus*. In China, the main types are cystic echinococcosis (CE) caused by the larva of *Echinococcus granulosus* and alveolar echinococcosis (AE) caused by the larva of *Echinococcus multilocularis*.

2.2 **Hydatidosis Endemic Area**

Areas where there is a cycle of *Echinococcus granulosus* or *Echinococcus multilocularis* between definitive hosts (such as dogs, foxes, wolves) and intermediate hosts (such as sheep, cattle, pigs, and other domestic and wild animals), and where human infection cases occur.

**3 Diagnostic Basis**

3.1 **Epidemiological History**

A history of residence, work, travel, or hunting in an endemic area, or contact with domestic animals such as dogs, cattle, sheep, or wild animals like foxes, wolves, and their fur; or, in non-endemic areas, a history of contact associated with transportation, slaughter, or processing of animal products and furs originating from endemic areas.

3.2 **Clinical Manifestations**

Patients with echinococcosis may be asymptomatic in the early stages, often discovered during physical examinations. The main clinical manifestations are a series of symptoms caused by compression, irritation, or rupture due to the space-occupying hydatid cyst. Cystic echinococcosis can occur in multiple organs throughout the body, most commonly the liver and lungs. The primary lesion of alveolar echinococcosis is

almost always in the liver, and most patients seeking medical attention are in the late stages. (See Appendix A)

3.3 **Imaging Examinations**

3.3.1 Detection of space-occupying lesions.

3.3.2 Identification of characteristic imaging features of echinococcosis in any of the following examinations (see Appendix B).

3.3.2.1 B-mode ultrasonography scan.

3.3.2.2 X-ray examination.

3.3.2.3 Computed Tomography (CT) or Magnetic Resonance Imaging (MRI) examination.

3.4 **Laboratory Examinations**

3.4.1 Detection of echinococcosis-specific antibodies, circulating antigens, or immune complexes in any of the following immunological tests (see Appendix C).

3.4.1.1 Enzyme-Linked Immunosorbent Assay (ELISA).

3.4.1.2 Indirect Hemagglutination Assay (IHA).

3.4.1.3 PVC Film Rapid-ELISA.

3.4.1.4 Immunoblotting (Western Blot, WB).

3.4.2 **Etiological Examination**: Detection of the hydatid cyst wall, daughter cysts, protoscoleces, or hooklets in surgical biopsy materials, resected lesions, or discharge (see Appendix D).

## 4 Diagnostic Principle

Comprehensive diagnosis based on epidemiological history, clinical manifestations, imaging characteristics, and laboratory test results.

## 5 Diagnostic Criteria

5.1 **Suspected Case**: Must simultaneously meet criteria 3.1 and 3.2, OR 3.1 and 3.3.1.

5.2 **Clinically Diagnosed Case**: A suspected case that meets criteria 3.3.2 OR 3.4.1.

5.3 **Confirmed Case**: A clinically diagnosed case that meets criterion 3.4.2.

## 6 Differential Diagnosis

6.1 **Differential Diagnosis of Hepatic Cystic Echinococcosis**

6.1.1 **Hepatic Cyst**: Imaging shows a thin cyst wall without the characteristic "double-layer wall" sign, and can be differentiated with echinococcosis immunological tests.

6.1.2 **Bacterial Liver Abscess**: Lacks characteristic imaging features of hydatid cysts. CT may show a low-density edema zone around the abscess wall; systemic toxic symptoms are more severe with significantly elevated white blood cell count; echinococcosis immunological tests are negative.

6.1.3 **Right Renal Pelvic Hydronephrosis and Gallbladder Hydrops**: Besides lacking characteristic hydatid cyst imaging features, echinococcosis immunological tests are negative.

6.2 **Differential Diagnosis of Hepatic Alveolar Echinococcosis**

6.2.1 **Hepatic Carcinoma**: Lesion progression is rapid with a relatively short course. Typical imaging shows the periphery of the lesion as mostly a "hypervascular area"; whereas the periphery of hepatic alveolar echinococcosis lesions is a "hypovascular area", with coexisting areas of solidification and liquefaction within the lesion. The lesion grows relatively slowly with a longer course. Differentiation can be effectively achieved using alpha-fetoprotein (AFP) and tumor-related biochemical tests, along with echinococcosis immunological tests.

6.2.2 **Hepatic Cystic Lesions**: Including congenital hepatic cysts and hepatic cystic echinococcosis. If hepatic alveolar echinococcosis is accompanied by a large liquefactive necrotic cavity, it can be misdiagnosed as a hepatic cyst or even hepatic cystic echinococcosis. Besides showing a liquefied cavity, imaging of alveolar echinococcosis also displays an irregular hyperechoic chamber wall or a "map sign" on ultrasound around its periphery. Congenital hepatic cysts have thinner walls with normal liver tissue appearance around them. Specific antigens for alveolar echinococcosis can be used to differentiate between hepatic cystic and alveolar echinococcosis.

6.2.3 **Bacterial Liver Abscess**: Same as 6.1.2.

# Appendix A

# (Informative Appendix)

# Epidemiology and Clinical Manifestations

## A.1 Epidemiology

Echinococcosis (hydatid disease) has a global distribution. Important endemic countries include China and Mongolia in East Asia; Turkey and Turkmenistan in Central Asia; Iraq, Syria, and Lebanon in West Asia; Argentina, Brazil, and Chile in South America; Australia and New Zealand in Oceania; and some countries in North, East, and Southern Africa.

In China, cystic echinococcosis (CE) predominates, mainly prevalent in pastoral and semi-pastoral areas of the northwest, with domestic dogs being the main source of infection and definitive host (harboring the adult tapeworm). China is one of the countries with a high prevalence of echinococcosis globally, with the seven provinces/regions of Xinjiang, Tibet, Ningxia, Gansu, Qinghai, Inner Mongolia, and Sichuan being the most severely affected. Alveolar echinococcosis (AE), also known as "worm cancer", is a highly fatal disease with a slightly more limited distribution, commonly seen in parts of Qinghai, Tibet, Gansu, Sichuan, Xinjiang, and Ningxia.

Echinococcosis can be divided into two types, cystic echinococcosis and alveolar echinococcosis, caused by the larvae of *Echinococcus granulosus*and *Echinococcus multilocularis*, respectively, and is an important zoonotic parasitic disease. When definitive hosts like domestic dogs pass mature proglottids containing numerous eggs, they contaminate grasslands, water sources, household environments, or attach to their fur. Herbivores and humans become infected by ingesting the eggs.

## A.2 Clinical Manifestations

Early stages may be asymptomatic, often discovered during physical examinations. The main clinical manifestations are a series of symptoms caused by compression, irritation, or rupture due to the space-occupying hydatid cyst. Cystic echinococcosis can

occur in multiple organs throughout the body, most commonly the liver and lungs. The primary lesion of alveolar echinococcosis is almost always located in the liver.

**A.2.1 Hepatic Cystic Echinococcosis**

A.2.1.1 **Manifestations of the space-occupying hydatid cyst**: Patients may present with hepatomegaly, a mass in the right upper quadrant, possible dull pain in the liver area, epigastric fullness, dyspepsia, weight loss, anemia, and portal hypertension. Dull persistent pain and tenderness in the liver area may be present. Infection of a hydatid cyst at the dome of the liver involving the diaphragm and pleura can cause adhesions, inflammatory infiltration, and right pleural effusion.

A.2.1.2 **Manifestations of hydatid cyst rupture**: Rupture into the peritoneal cavity is most common and can lead to secondary abdominal echinococcosis. Most patients may experience allergic reactions, showing skin erythema, pruritus, urticaria, nausea, chest tightness, etc. A few may have severe anaphylactic shock. Patients may suddenly experience upper abdominal pain, which can involve the entire abdomen, resembling perforation of a hollow viscus, but may resolve or even disappear spontaneously within tens of minutes. On physical examination, the patient may only have tenderness in the upper abdomen without significant muscle guarding elsewhere, but peritoneal irritation signs are more obvious if the ruptured cyst is infected or has a biliary fistula. Anaphylactic shock is often a severe consequence of cyst rupture and is one of the main causes of death.

**A.2.2 Hepatic Alveolar Echinococcosis**: Mainly presents as dull pain in the upper abdomen, sometimes accompanied by colicky abdominal pain and chills with high fever indicating infection; hepatomegaly or a palpable mass in the liver area, the liver is firm and sometimes hard nodules can be felt; varying degrees of cholestatic jaundice and signs of portal hypertension. The alveolar hydatid has an "invasive growth" characteristic similar to liver cancer, can metastasize, and cause symptoms related to the organs where metastases occur. Main complications include sepsis or toxic shock due to obstruction and infection of the biliary system, impaired liver function, leading to liver failure or multiple organ failure and death.

**A.2.3 Pulmonary Cystic Echinococcosis**: May present with chest pain (dull or distending) or irritating cough. Large cysts can cause compressive atelectasis, leading to

chest tightness, shortness of breath, or even dyspnea. Secondary infection can cause symptoms of lung abscess, fever, chest pain, cough with purulent sputum; if a bronchial fistula is present, cyst debris may be present in the sputum, and severe cases may involve hemoptysis. Rupture into a bronchus causes violent coughing with expectoration of large amounts of watery cyst fluid containing fragments of the inner cyst wall, and in severe cases, death by asphyxiation. Occasionally, a patient may cough up the entire hydatid cyst contents, leading to collapse and closure of the pericyst, resulting in a cure. However, complete expulsion is rare, the cavity often becomes secondarily infected, the surrounding lung tissue develops chronic inflammation, and surgical treatment is usually indicated. Rupture into the pleural cavity causes hydropneumothorax or pyopneumothorax, followed by secondary multiple pleural cystic echinococcosis.

**A.2.4 Echinococcosis at Other Sites**: Cystic echinococcosis can occur in the abdominal and pelvic cavities, spleen, kidney, brain, bone, mediastinum, heart, muscle and skin, bladder, ovary, testis, eye, etc. Alveolar echinococcosis can metastasize to lungs, brain, etc., causing corresponding localized space-occupying symptoms and signs of compression, irritation, or allergic reactions. A few patients may have mixed infections with both types of echinococcosis. Individual patients with alveolar echinococcosis may present with parasitic embolism.

# Appendix B
# (Normative Appendix)
# Characteristic Imaging of Echinococcosis

## B.1 Characteristic B-mode Ultrasound Imaging of Echinococcosis

B.1.1 **Ultrasound Imaging Diagnosis and Classification of Cystic Echinococcosis**: Cystic echinococcosis is classified into six types on B-mode ultrasound imaging: cystic lesion (CL type), unilocular cyst (CE type I), multilocular cyst with daughter cysts (CE type II), cyst with detached endocyst (CE type III), solid/calcified cyst (CE type IV), and calcified cyst (CE type V).

B.1.1.1 **Cystic Lesion (CL)**: Ill-defined cyst wall containing homogeneous echogenic content, generally round or oval.

B.1.1.2 **Unilocular Cyst (CE I)**: The hydatid cyst is filled with watery fluid, appearing as a round or oval anechoic area. The distinct difference in density between the cyst wall and liver tissue results in a clearly demarcated wall. A specific feature is the potential space interface between the inner germinal layer and outer laminated layer, which may show the "double-wall sign". Ultrasound shows obvious posterior enhancement. When the probe vibrates the cyst, small floating echoes may be seen within the anechoic area, called the "hydatid sand" feature.

B.1.1.3 **Multilocular Cyst with Daughter Cysts (CE II)**: Multiple smaller spherical anechoic shadows and hyperechoic rings can be seen within the anechoic maternal cyst, forming the characteristic "cyst within cyst" or "rosette" appearance. Ultrasound or CT may show a "honeycomb" or "wheel-spoke" pattern.

B.1.1.4 **Cyst with Detached Endocyst (CE III)**: Endocyst detachment: After rupture of the hepatic hydatid cyst, fluid enters between the endocyst and ectocyst, showing the "water-lily sign" or "floating membrane sign"; if part of the cyst wall detaches from the outer wall, it shows the "floating membrane sign" or "serpent sign", then the wall collapses, inverts, curls, and floats in the fluid, showing the "water-lily sign" or "serpent sign".

B.1.1.5 **Solid/Calcified Cyst (CE IV)**: The hydatid cyst gradually degenerates and dies, fluid is absorbed, the wall folds and contracts, followed by caseous necrosis. Ultrasound shows a heterogeneous pattern with mixed hypo- and hyperechoic areas resembling "gyri of the brain".

B.1.1.6 **Calcified Cyst (CE V)**: Long disease course, the outer laminated layer of the hydatid becomes thickened, rough, and calcified, sometimes completely calcified. Ultrasound shows increased and heterogeneous echogenicity of the cyst, with a thickened, flocculent wall accompanied by broad posterior acoustic shadowing and lateral shadowing.

B.1.2 **Imaging Diagnosis and Classification of Alveolar Echinococcosis**:

B.1.2.1 **Infiltrative Type**: Ultrasound shows hepatomegaly, detection of an echogenic mass with mixed low and high echogenicity, blurred peripheral borders, and posterior acoustic attenuation.

B.1.2.2 **Calcified Lesion Type**: During the process of *E. multilocularis*metacestode eroding liver tissue, calcium salt deposition occurs within the alveolar echinococcosis lesion. Punctate calcified granules appear early. As the disease progresses, calcified granules merge into flocculent or irregular large calcified plaques. Ultrasound shows a space-occupying lesion with mixed low to medium echogenicity in the liver, containing scattered calcified spots or irregular large calcified hyperechoic plaques with acoustic shadowing.

B.1.2.3 **Lesion with Liquefactive Cavity Type**: The multilocular hydatid proliferates into a massive lesion; its center undergoes ischemic necrosis, liquefying into a gelatinous substance, forming an irregular necrotic liquefactive cavity. Ultrasound shows a large, irregular anechoic liquid area within a heterogeneous hyperechoic mass, with posterior echo enhancement, showing the "cavity sign".

**B.2 Characteristic X-ray Imaging of Echinococcosis**

B.2.1 **Pulmonary Cystic Echinococcosis**: Hydatid cysts less than 2 cm in diameter appear as spherical shadows of low density with rough, blurred edges. Larger cysts have clear contours, smooth edges, sharp margins, uniform density, and appear as round, oval, or notched/lobulated, solitary or multiple cystic shadows. Displacement of the trachea or

heart may occur due to compression by the cyst. Hydatid cysts in the lower lung lobes may exhibit shape changes with respiration ("hydatid respiratory sign").

B.2.2 **Hepatic Cystic Echinococcosis**: Plain abdominal film shows enlargement of the liver contour; a cyst at the dome of the liver elevates the right diaphragm or protrudes into the thoracic cavity. Large cysts can elevate the diaphragm, reduce respiratory mobility, and even displace the heart to the left. Cysts in the mid-to-lower liver do not significantly elevate the diaphragm; a hemispherical shadow of increased density may be seen at the lower liver edge. Calcified cysts show calcified shadows.

**B.3 Characteristic Computed Tomography (CT) Imaging of Echinococcosis**

B.3.1 **Hepatic Cystic Echinococcosis**: Larger cysts cause enlargement of the liver contour, showing spherical or oval cystic space-occupying shadows of varying sizes within the liver parenchyma. The inner cyst wall is smooth, 1-3 mm thick, CT value 30-40 HU. The cyst is filled with fluid of water density, CT value 10-20 HU. The outer cyst wall is thicker, 3-8 mm, and may show the "double-wall sign", CT value 30-50 HU, with clear boundaries. On contrast enhancement, surrounding liver tissue density increases while the cyst density does not, clearly showing the boundary, aiding differentiation from hemangioma and liver cancer. Daughter cyst fluid has lower density than maternal cyst fluid. When daughter cysts are present, multiple smaller round low-density shadows with slightly lower density are seen. An excessive number of daughter cysts can fill the mother cyst, pressing against each other into square, rhomboid, or honeycomb shapes. Calcified outer cyst appears as an irregular "eggshell" structure, or as plaques, strips, or the entire cyst may be calcified, CT value >60 HU. After endocyst rupture, the cyst wall collapses forming various irregular images. Due to death of the parasite, cyst fluid is absorbed and concentrated, resembling caseous degeneration and containing degenerated daughter cysts, with increased and heterogeneous CT values, similar to a solid tumor image, but no enhancement on CT contrast scan. Cysts located at the dome or edge of the liver may show a spherical or semi-arcuate protruding border.

B.3.2 **Hepatic Alveolar Echinococcosis**: CT scan shows irregular, inhomogeneous low-density shadows, density lower than liver tissue. Contrast enhancement shows no enhancement effect in the lesion, forming a characteristic "hypovascular area", which can differentiate it from the "hypervascular area" of hemangioma or liver cancer lesions. A

low-density "infiltrative zone" can be seen due to expansion of the alveolar hydatid towards the periphery. During the degenerative process accompanied by calcium salt deposition, a "calcified zone" appears. Shows a high-density calcified lesion with low-density fluid-filled cavities of various sizes and irregular shapes. The liquefied area is surrounded by a calcified wall forming the "rock cave sign". Large calcified shadows at the edge of the liquefied area may protrude into it, showing the "peninsula sign". Alveolar echinococcosis continuously invades and proliferates into surrounding liver tissue, forming the "small vesicle sign", indicating fresh lesions; no enhancement on contrast scan. The appearance of multiple concentric, fine granular calcifications within the lesion is characteristic of fresh alveolar echinococcosis lesions, with no enhancement on contrast scan. The lesion's CT manifestations: irregular lesions showing low signal on both T1- and T2-weighted MRI images. Internal necrosis forms liquefaction cavities. Small new vesicles at the periphery remain viable, proliferating and expanding, eroding liver tissue ("halo sign"). Because the cavity wall is composed of thickened fibrous tissue forming the boundary, irregular in shape, MRI can show the cavity wall as low signal on both T1WI and T2WI, with the peripheral infiltrative zone showing low signal, the "map sign".

# Appendix C
# (Normative Appendix)
# Immunological Diagnostic Methods

Immunological diagnostic methods for human echinococcosis include Indirect Hemagglutination Assay (IHA), Enzyme-Linked Immunosorbent Assay (ELISA), PVC Film Rapid-ELISA, etc. Among these, the ELISA method is the most commonly used and relatively sensitive. Existing immunological tests for echinococcosis vary greatly in sensitivity and specificity. Test results are influenced by many factors: the nature and quality of the antigen; the test system used; the number, location, and viability of hydatid cysts; differences in geographical strains; and individual immune response differences. Approximately 10-40% of surgically confirmed echinococcosis patients cannot be detected for specific antibodies using currently known antigens. This section lists some commonly used methods internationally and domestically.

**C.1 Antigen Preparation**

C.1.1 **Crude Hydatid Fluid Antigen**: After cleaning and disinfecting the surface of an intact hydatid cyst, aspirate cyst fluid aseptically (preferably from fertile cysts without suppuration or calcification). Centrifuge the cyst fluid at 5000 r/min for 20 minutes, store the supernatant frozen for later use.

C.1.2 **Purified Antigen**

Phosphotungstic acid and magnesium chloride precipitation can be used for partial purification of hydatid cyst fluid antigen. Take 20 ml of centrifuged cyst fluid supernatant, add 1.2 ml each of 2 mol/L $MgCl_2$ and 4% phosphotungstic acid (pH adjusted to 7.5-7.6 with NaOH); stir at room temperature for 5 min, centrifuge at 5000 r/min for 30 min; dissolve the precipitate in 0.02 mol/L pH 7.2 PBS; dialyze at 4°C for 24 hours, measure protein concentration, store frozen at -20°C.

**C.2 Enzyme-Linked Immunosorbent Assay (ELISA)**

C.2.1 **Antigens**

Crude hydatid fluid antigen, purified antigen, specific antigen.

C.2.2 **Operational Procedure**

C.2.2.1 **Antigen Coating**: Dilute antigen to working concentration with 0.05 mol/L pH 9.6 carbonate buffer, add 100 μl per well, place in a humid chamber at 4°C overnight. The next day, discard antigen, wash 3 times with phosphate-buffered saline containing 0.05% Tween-20 (0.02 mol/L, pH 7.4 PBST), 3 min each time, pat dry;

C.2.2.2 **Add Test Serum**: Dilute serum 1:200 with PBST containing 3% BSA, add 100 μl per well. Each plate should include one reference positive well, three reference negative wells, and one PBS control well. Place in humid chamber, 37°C for 1 h, remove and discard serum, wash 3 times as before;

C.2.2.3 **Add Enzyme-Labeled Secondary Antibody**: Add 100 μl of working concentration Horseradish Peroxidase (HRP)-labeled anti-human IgG, 37°C, 1 h, discard secondary antibody, wash as before;

C.2.2.4 **Add Substrate for Chromogenic Reaction**: Add 100 μl of O-phenylenediamine (OPD, orange-red) or tetramethylbenzidine/tetramethylbenzidine sulfate (TMB/TMBS, blue) substrate solution, 37°C, 30 min;

C.2.2.5 **Add Stop Solution**: 50 μl of 2 mol/L $H_2SO_4$.

C.2.3 **Result Interpretation**

Read absorbance at 492 nm (OPD) or 450 nm (TMB/TMBS) on a microplate reader. A sample (S) to negative control (N) ratio (S/N) ≥ 2.1 is considered the positive cut-off value.

**C.3 Indirect Hemagglutination Assay (IHA)**

C.3.1 **Preparation of Aldehyde-Fixed Sheep Red Blood Cells**

C.3.1.1 Aseptically collect 100 ml of sheep blood from the jugular vein, add to 200-400 ml of Alsever's solution (dissolve 8.2 g glucose, 1.68 g NaCl, 2.4 g sodium citrate, 0.22 g citric acid in 400 ml triple-distilled water, adjust pH to 6.1, autoclave or filter sterilize), mix for anticoagulation. Centrifuge at 3000 r/min for 15 min, discard supernatant;

C.3.1.2 Wash red blood cells 3 times with physiological saline, centrifuge as above. Add 50 ml physiological saline and 100 ml PBS (0.15 mol/L pH 8.0) to the pellet, mix;

C.3.1.3 Add 300 ml of glutaraldehyde neutralized with 10% $Na_2CO_3$ (mix 75 ml 10% $Na_2CO_3$ with 225 ml 25% glutaraldehyde), stir slowly on a magnetic stirrer at 4°C for 24 h;

C.3.1.4 Centrifuge, discard supernatant, wash pellet with physiological saline 5 times. Finally, resuspend the red blood cell pellet in physiological saline containing 0.1% $NaN_3$ to make a 10% cell suspension, store at 4°C.

C.3.2 **Sensitization of Red Blood Cells**

C.3.2.1 Wash 20 ml of 10% glutaraldehyde-fixed sheep red blood cells 3 times with 300 ml physiological saline (3000 r/min, 5 min), the packed cell volume is approximately 2 ml;

C.3.2.2 Add 98 ml physiological saline to make 100 ml of a 2% red blood cell suspension;

C.3.2.3 Mix with 100 ml of 1:10000 tannic acid, incubate in a 37°C water bath for 30 min, mix by vortexing every 10 min;

C.3.2.4 Centrifuge and wash 3 times as in C.3.2.1, discard supernatant;

C.3.2.5 Add 198 ml physiological saline to the pellet to make 200 ml of a 1% tanned red blood cell suspension;

C.3.2.6 Mix with 200 ml of diluted cyst fluid antigen, incubate in a 37°C water bath for 30 min, mix by vortexing every 10 min;

C.3.2.7 Centrifuge, discard supernatant, wash 3 times with 1% rabbit serum PBS (0.15 mol/L pH 7.2 PBS), discard supernatant;

C.3.2.8 Resuspend red blood cells in 18 ml of 10% rabbit serum PBS to make 20 ml of a 10% sensitized red blood cell suspension. Store at 4°C.

C.3.3 **Operational Procedure**

C.3.3.1 **Prepare Antigen**: Dilute the 10% sensitized red blood cell stock solution to 1% with 0.15 mol/L pH 7.2 PBS for use;

C.3.3.2 Add 25 μl PBS to each well of the hemagglutination plate;

C.3.3.3 Add 25 μl of test serum to the first well, serially dilute from the first well backward using a pipette or diluter;

C.3.3.4 Add 25 μl of 1% sensitized red blood cells to each well, mix by shaking for 3 min, incubate at 37°C for 1 h, then read results;

C.3.3.5 **Control Setup**: Each test batch should include positive, negative, and red blood cell controls (red blood cell control well contains only PBS and sensitized red blood cells).

C.3.4 **Result Interpretation**

C.3.4.1 **Negative Reaction**: All red blood cells settle at the bottom of the well as a compact dot with a smooth edge;

C.3.4.2 **Positive Reaction**: ++++ 100% red blood cell agglutination; +++ 75% red blood cell agglutination; ++ 50% red blood cell agglutination; + 25% red blood cell agglutination.

A serum dilution of 1:128 showing a positive reaction (++) is judged as serum antibody positive.

**C.4 PVC Film Rapid-ELISA**

C.4.1 **Antigens**

Crude hydatid fluid antigen, purified antigen, specific antigen.

C.4.2 **Operational Procedure**

C.4.2.1 Take the PVC film soft plate pre-coated with antigen, number it, wash once with PBST, then add 200 µl PBST to each well;

C.4.2.2 Add test serum and reference sera (one negative control and one positive control per plate) 10 µl per well, mix, place in a humid chamber at 37°C for 5 min (or at 25°C room temperature for 10 min). Discard serum, wash 8 consecutive times with PBST, shake dry;

C.4.2.3 **Add Enzyme-Labeled Antibody**: Add 200 µl of working concentration enzyme-labeled antibody to each well, 37°C for 5 min, wash 8 times as above, then wash once with distilled water, shake dry;

C.4.2.4 **Add Substrate for Chromogenic Reaction**: Add 200 µl TMB substrate to each well, react for 5-10 min (Preparation of TMB substrate solution: Dissolve 50 mg TMB in 10 ml dimethyl sulfoxide as stock solution, store at 4°C. Before use, take 1 ml stock solution + 50 ml pH 5.0 citrate buffer + 8 µl 30% $H_2O_2$).

C.4.3 **Result Interpretation**

Judge results visually by comparison with negative and positive controls. Negative is essentially colorless, positive is bright blue.

**C.5 Immunoblotting (Western Blot, WB)**

C.5.1 **Antigen Membrane Preparation**

Perform conventional SDS-PAGE gel electrophoresis on crude hydatid fluid antigen, crude protoscolex antigen, or specific purified antigen, with a separation gel concentration of 15%. After antigen proteins are separated by SDS-PAGE electrophoresis, transfer them onto a nitrocellulose membrane. Stain with Ponceau S, mark standard molecular weight positions. Block the antigen membrane with blocking solution (3% skimmed milk powder, 0.15 mol/L NaCl, 0.05 mol/L Tris-HCl, pH 7.4, 0.02% Tween-20) for 1 h, then cut the membrane into strips 2-3 mm wide for use. These antigen membrane strips can be sandwiched between PBS-moistened filter paper, sealed in a plastic bag, and stored at 4°C. If stored in a low-temperature freezer, they can be preserved long-term.

C.5.2 **Operational Procedure**

C.5.2.1 Place the antigen membrane in a reaction trough, add test serum diluted 1:100 with blocking solution (3% skimmed milk powder, 0.15 mol/L NaCl, 0.05 mol/L Tris-HCl, pH 7.4, 0.02% Tween-20). Each batch of tests should include reference positive, negative, and PBS controls. Shake at room temperature for 2 h (can be overnight at 4°C). The next day, wash 4 times with the above blocking solution, 5 min each time;

C.5.2.2 Add working concentration Horseradish Peroxidase (HRP)-labeled anti-human IgG, incubate at room temperature for 2 h, wash as before;

C.5.2.3 Add 3,3'-Diaminobenzidine (DAB, brown) for color development until clear, stop the reaction with distilled water, dry and store the reaction membrane.

C.5.3 **Result Interpretation**

Specific reaction bands for *E. granulosus*: 8 kDa, 16 kDa, 20-24 kDa (AgB) and 38 kDa (Ag5)

Specific reaction bands for *E. multilocularis*: 18 kDa (Em18), 54 kDa (Em2) and 220 kDa (EmAP)

**C.6 Circulating Antigen (cAg) Detection**

C.6.1 **Preparation of Specific Monoclonal or Polyclonal Antibodies**

C.6.1.1 Immunize Balb/c mice with crude or specific antigen, perform cell fusion, positive cell clone screening, and identification using conventional methods. Inject the cell line secreting specific monoclonal antibodies intraperitoneally into mice to obtain monoclonal antibody ascites;

C.6.1.2 Immunize rabbits with crude or specific antigen to obtain high-titer polyclonal antibodies;

C.6.1.3 Establish a sensitive double antibody sandwich ELISA method capable of detecting crude or specific antigens;

C.6.1.4 Use the established double antibody sandwich ELISA system to detect cAg in patient serum.

C.6.2 **Circulating Antigen Detection**

C.6.2.1 Coat the microplate with specific monoclonal or polyclonal antibody diluted to working concentration in 0.05 mol/L pH 9.6 carbonate buffer, 100 µl per well, 4°C overnight, then wash plate 3 times with PBST;

C.6.2.2 Add 100 µl PBST containing 3% BSA to each well, block at 37°C for 1 h, wash plate 3 times;

C.6.2.3 Dilute test serum 1:2 with the above solution, add 100 µl per well, incubate at 37°C for 1 h, wash plate 3 times;

C.6.2.4 Add 100 µl of working concentration enzyme-labeled secondary antibody (specific monoclonal or polyclonal antibody), incubate at 37°C for 1 h, then wash plate 3 times;

C.6.2.5 Add 100 µl of O-phenylenediamine (OPD, orange-red) or tetramethylbenzidine/tetramethylbenzidine sulfate (TMB/TMBS, blue) substrate solution, incubate at 37°C for 30 min;

C.6.2.6 Add 50 µl of 2 mol/L $H_2SO_4$ to stop the reaction;

C.6.2.7 Measure absorbance of each well with a microplate reader;

C.6.2.8 **Control Setup and Result Judgment**: Each plate should have one positive control well (100 µl of 1 µg/ml antigen), three negative serum control wells. The positive cut-off value is the mean OD of negative controls + 3SD or S/N ≥ 2.0.

**C.7 Circulating Immune Complex (CIC) Detection**

C.7.1 **Detection of Circulating Antigen within CIC**

C.7.1.1 Prepare a 7% solution of PEG 6000 with 0.01 mol/L pH 8.4 borate buffer. Take 100 μl of this solution and mix with an equal volume of 1:2 diluted test serum, let stand at room temperature for 1 h, then centrifuge at 5000 r/min for 1 h;

C.7.1.2 Aspirate the supernatant, add 0.5 ml of 0.05 mol/L pH 9.6 carbonate buffer containing 8 mol/L urea to the precipitate to dissolve it;

C.7.1.3 Use this solution to coat the microplate, 100 μl per well, coat two wells per specimen. Incubate at 4°C overnight, then wash plate 3 times (treat positive and negative controls similarly);

C.7.1.4 Add 100 μl PBST containing 3% BSA to each well, block at 37°C for 1 h, wash plate 3 times;

C.7.1.5 Add 100 μl of working concentration HRP-labeled specific monoclonal or polyclonal antibody, incubate at 37°C for 1 h, then wash plate 3 times;

C.7.1.6 Add 100 μl of O-phenylenediamine (OPD, orange-red) or tetramethylbenzidine/tetramethylbenzidine sulfate (TMB/TMBS, blue) substrate solution, incubate at 37°C for 30 min.

C.7.1.7 Add 50 μl of 2 mol/L $H_2SO_4$ to stop the reaction;

C.7.1.8 Measure absorbance of each well with a microplate reader;

C.7.1.9 **Control Setup and Result Judgment**: Each plate should have one positive control well (100 μl of 1 μg/ml antigen), three negative serum control wells. The positive cut-off value is the mean OD of negative controls + 3SD.

C.7.2 **Detection of CIC**

C.7.2.1 Preparation of specific antibodies is the same as C.6.1;

C.7.2.2 Coat the microplate with specific monoclonal or polyclonal antibody diluted to working concentration in 0.05 mol/L pH 9.6 carbonate buffer, 100 μl per well, 4°C overnight, then wash plate 3 times with PBST;

C.7.2.3 Add 100 μl PBST containing 3% BSA to each well, block at 37°C for 1 h, wash plate 3 times;

C.7.2.4 Dilute test serum 1:20 with the above solution, add 100 μl per well, incubate at 37°C for 1 h, wash plate 3 times;

C.7.2.5 Add 100 μl of working concentration enzyme-labeled anti-human IgG or IgM, incubate at 37°C for 1 h, then wash plate 3 times;

C.7.2.6 Add 100 μl of O-phenylenediamine (OPD, orange-red) or tetramethylbenzidine/tetramethylbenzidine sulfate (TMB/TMBS, blue) substrate solution, incubate at 37°C for 30 min;

C.7.2.7 Add 50 μl of 2 mol/L $H_2SO_4$ to stop the reaction;

C.7.2.8 Measure absorbance of each well with a microplate reader;

C.7.2.9 **Control Setup and Result Judgment**: Each plate should have one positive control well (artificial complex), three negative serum control wells. A positive result is indicated by $S/N \geq 2.0$.

# Appendix D
# (Normative Appendix)
# Parasitological and Histopathological Examinations

**D.1 Echinococcus granulosus**

Appears cystic, containing fluid, round or oval, mostly unilocular, diameter ranges from less than 1 cm to over 10 cm, with giant cysts reaching 30 cm. Histological examination shows the cyst wall divided into two layers: an outer acellular laminated layer and an inner germinal layer. The germinal layer gives rise inwards to numerous protoscoleces or brood capsules.

Pulmonary echinococcosis patients may cough up sputum containing hydatid cyst wall fragments, daughter cysts, protoscoleces, and rostellar hooks after cyst rupture. Cyst wall and daughter cysts can be identified macroscopically, but histological examination should still be performed. Sputum can be examined directly by smear microscopy. It is preferable to dilute the sputum, centrifuge, and examine the sediment. Liver echinococcosis patients can be examined by fine-needle aspiration under ultrasound guidance, or material obtained after surgical removal of the hydatid can be examined.

**D.2 Echinococcus multilocularis**

Typical *E. multilocularis*metacestode consists of countless small, irregular vesicles less than 1 mm to 30 mm in diameter forming an alveolar structure. Due to degeneration and necrosis, a liquefactive cavity filled with necrotic material often forms in the center of the lesion. Microscopic examination reveals a thin PAS-positive laminated layer; the germinal layer is often difficult to discern. The alveolar hydatid in human infection rarely forms brood capsules and protoscoleces. The interior of the alveolar hydatid is the necrotic area, surrounded by an infiltrate of histiocytes and lymphocytes. There is a chronic inflammatory reaction, tissue fibrosis, and calcification around the alveolar hydatid. Tissue fibrosis makes the alveolar hydatid dense and hard.

**References**


1. WHO/OIE Manual on Echinococcosis in Humans and Animals: a Public Health Problem of Global Concern. Edited by J. Eckert, M. A. Gemmell, F.-X. Meslin and Z. S. Pawlowski. World Organisation for Animal Health (Office International des Epizooties) and World Health Organization, 2001. Reprinted: January 2002.
2. Craig P, Pawlowski Z. Cestode Zoonoses: echinococcosis and cysticercosis. An emergent and global problem. IOS Press.
3. Oriol R, Williams J F. Purification of lipoprotein antigens of *Echinococcus granulosus*from sheep hydatid fluid. Am J Trop Med Hyg 1971;20:569-574.
4. Gottstein B, Eckert J, Fey H. Serological differentiation between *Echinococcus granulosus*and *Echinococcus multilocularis*infections in Man. Parasitol Res, 1983;69:347-356.
5. Ito A., Nakao M., Kutsumi H. Serodiagnosis of alveolar hydatid disease by Western Blotting. Trans R Soc Trop Med Hyg 1993;87:170-172.
6. Jiang L, Wen H and Ito A. Immunodiagnostic differentiation of alveolar and cystic echinococcosis using ELISA test with 18-kDa antigen extracted from Echinococcus protoscoleces. Trans Roy Soc Trop Med Hyg 2001;95(3):285-288.

**People's Republic of China**

**Health Industry Standard**

**Diagnostic Criteria for Echinococcosis**

**WS 257—2006**

Published by: People's Medical Publishing House (China Main Line: 010 - 67616688)

Address: Building 3, Fangzhuang Qunguan Community, Fengtai District, Beijing

Postcode: 100078

Website: http://www.pmph.com

E-mail: pmph@pmph.com

Purchase Hotline: 010 - 67605754 or 010 - 65264830

Printed by: Beijing Xinfeng Printing Factory

Distributed by: Xinhua Bookstore

Format: 880×1230 1/16

Sheet: 1.25

Word Count: 28,000 words

Edition: 1st Edition, December 2006

Printing: 1st Printing of the 1st Edition, December 2006

Book Number: 14117·61
